# SOX10 ablation severely impairs the generation of postmigratory neural crest from human pluripotent stem cells

**DOI:** 10.1038/s41419-021-04099-4

**Published:** 2021-08-27

**Authors:** Xingqiang Lai, Jia Liu, Zhengwei Zou, Yina Wang, Ye Wang, Xiao Liu, Weijun Huang, Yuanchen Ma, Qian Chen, Fugui Li, Guifu Wu, Weiqiang Li, Weijia Wang, Yong Yuan, Boxiong Jiang

**Affiliations:** 1 Department of Cardiology, The Eighth Affiliated Hospital, Sun Yat-sen University, Shenzhen, Guangdong China; 2 Center for Stem Cell Biology and Tissue Engineering, Key Laboratory for Stem Cells and Tissue Engineering, Ministry of Education, Zhongshan School of Medicine, Sun Yat-Sen University, Guangzhou, Guangdong China; 3 VIP Medical Service Center, The Third Affiliated Hospital of Sun Yat-sen University, Guangzhou, China; 4 Center for Stem Cell Clinical Translation, First Affiliated Hospital, Gannan Medical University, Ganzhou, Jiangxi China; 5 Fetal Medicine Center, Department of Obstetrics and Gynecology, The First Affiliated Hospital, Sun Yat-sen University, Guangzhou, China; 6 Department of Laboratory Medicine, Zhongshan People’s Hospital, Zhongshan, Guangdong China; 7 NHC Key Laboratory of Assisted Circulation, The First Affiliated Hospital of Sun Yat-Sen University, Guangzhou, China; 8 Department of Cardiovascular Center, Zhongshan People’s Hospital, Zhongshan, Guangdong China

**Keywords:** Differentiation, Multipotent stem cells

## Abstract

Animal studies have indicated that SOX10 is one of the key transcription factors regulating the proliferation, migration and differentiation of multipotent neural crest (NC), and mutation of *SOX10* in humans may lead to type 4 Waardenburg syndrome (WS). However, the exact role of SOX10 in human NC development and the underlying molecular mechanisms of SOX10-related human diseases remain poorly understood due to the lack of appropriate human model systems. In this study, we successfully generated SOX10-knockout human induced pluripotent stem cells (SOX10^−/−^ hiPSCs) by the CRISPR-Cas9 gene editing tool. We found that loss of SOX10 significantly inhibited the generation of p75^high^HNK1^+^/CD49D^+^ postmigratory neural crest stem cells (NCSCs) and upregulated the cell apoptosis rate during NC commitment from hiPSCs. Moreover, we discovered that both the neuronal and glial differentiation capacities of SOX10^−/−^ NCSCs were severely compromised. Intriguingly, we showed that SOX10^−/−^ hiPSCs generated markedly more TFAP2C^+^nonneural ectoderm cells (NNE) than control hiPSCs during neural crest differentiation. Our results indicate that SOX10 is crucial for the transition of premigratory cells to migrating NC and is vital for NC survival. Taken together, these results provide new insights into the function of SOX10 in human NC development, and the SOX10-knockout hiPSC lines may serve as a valuable cell model to study the pathogenesis of SOX10-related human neurocristopathies.

## Introduction

Neural crest stem cells (NCSCs) are multipotent cells in the developing vertebrate embryo that give rise to a wide range of tissues and cell types, including the peripheral nervous system (PNS), enteric nervous system (ENS), craniofacial skeletal tissue, and melanocytes of the skin [1, 2]. It was reported that migration, proliferation, or differentiation defects of NCSCs account for ~30% of all congenital malformations in humans [3]. Although neural crest development has been systematically studied in a variety of animal models, including zebrafish, chicks and mice [4–7], these models may not be ideal representations of normal human development or disease phenotypes due to differences in anatomy, physiology, pathophysiology, and genetic background. In recent years, there have also been numerous efforts to study neural crest development and their related diseases using human pluripotent stem cell (hPSC) models [8–11]. Several protocols have been established for the robust differentiation of NCSCs from hPSCs [12–15]. These methods provide us with valuable platforms for studying the cellular and molecular mechanisms involved in human NCSC development and neurocristopathies.

SRY-Box Transcription Factor 10 (*SOX10*) encodes a 466-amino-acid protein that contains a DNA-binding motif known as the high-mobility group (HMG) domain and a transactivation domain [16, 17]. SOX10 is expressed in NCSCs and their melanoblastic and glial derivatives [18], and it is involved in the regulation of neural crest development and determination of cell fate [19]. SOX10 deficiency leads to severe defects in NC derivatives, including dorsal root ganglia (DRG) sensory neurons, enteric and autonomic neurons, melanocytes and peripheral glial cells [20]. It was reported that homozygotes of Dominant megacolon mice with *SOX10* mutation (*SOX10*^Dom/Dom^) die in utero prior to 13 days of gestation, and a significant proportion of heterozygous Dom mice were lost before weaning because of the occurrence of megacolon [18, 21], while SOX10-knockout mice (*SOX10*^Lacz/Lacz^) have phenotypes similar to those of Dom mice [22]. More importantly, mutations in the human *SOX10* gene are associated with several neurocristopathies, including demyelinating disorders and type 4 Waardenburg disease (WS4), which is characterized by pigmentary disturbances, hearing impairment, and intestinal aganglionosis [23–25]. Although SOX10 was shown to preserve the self-renewal and multipotency of NCSCs via interaction with the Neuregulin-1, BMP2/4, and TGFβ pathways in rodent models [18, 26], the role of SOX10 in human NC development and the underlying pathogenesis of SOX10-related human diseases have not yet been fully elucidated due to the lack of ideal human model systems.

In this study, we successfully established SOX10-knockout human induced pluripotent stem cell (hiPSC) lines using a CRISPR/Cas9-mediated gene disruption system [27, 28]. We discovered that loss of SOX10 significantly inhibited the development of migrating NCSCs from premigratory NCSCs, upregulated the cell apoptosis rate, and promoted the development of nonneuroectoderm during in vitro neural crest commitment of hiPSCs. Moreover, we found that the neuronal and glial differentiation capacities of *SOX10*^−/−^ NCSCs were severely compromised.

## Materials and methods

### Cell culture

Human pluripotent stem cells, including human embryonic fibroblast-derived hiPSCs (HEF-hiPSCs; designated WT1) and H9 cells (a human embryonic stem cell line; designated WT2) [29], were maintained on Matrigel Growth Factor-Reduced Basement Membrane Matrix (BD Biosciences, San Diego, CA, USA)-coated 6-well plates in mTeSR1 medium (Stem Cell Technologies, Vancouver, Canada). The culture medium was replaced daily. Cells were passaged every 4–5 days using 0.5 mM EDTA (Invitrogen, Carlsbad, CA, USA) at a split ratio of 1:6.

### Plasmid construction

**Fig. 1:**
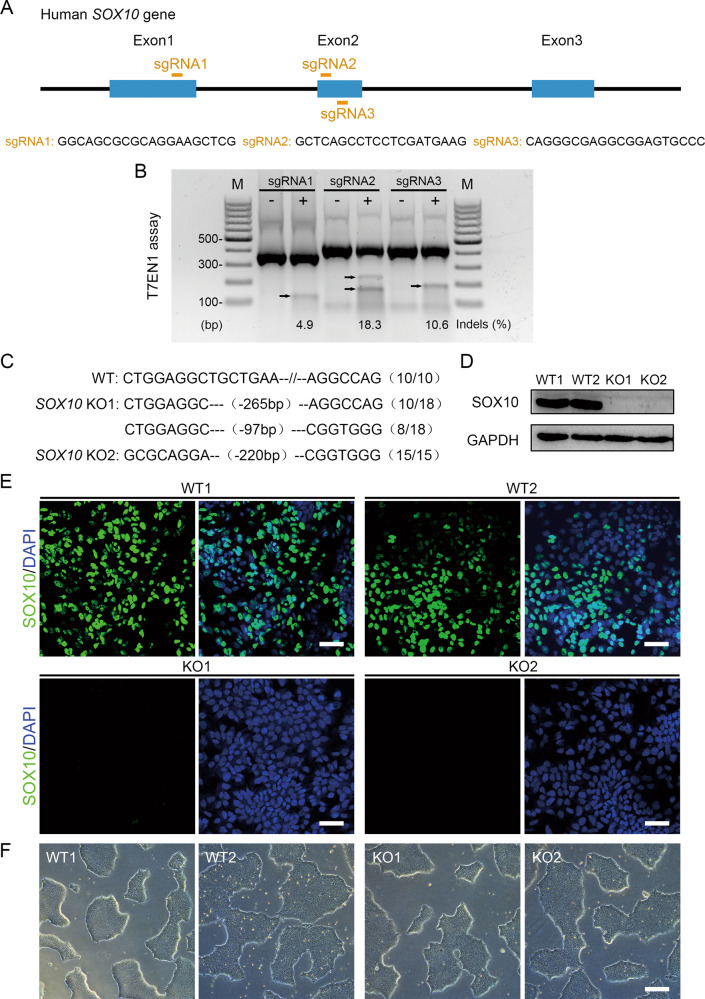
Generation and verification of SOX10-knockout hiPSCs. **A** Schematic diagram of sgRNAs targeting at exons 1 and 2 of the human *SOX10* locus. **B** Detection of sgRNA:Cas9-mediated cleavage of *SOX10* by PCR and T7EN1 cleavage assay (M, DNA marker). **C** Sanger sequencing was performed on PCR products amplifed from SOX10-knockout hiPSCs. Deletions (−). N/N indicates positive colonies out of total sequenced. WT wild-type sample, KO1 and KO2 the two mutant samples. **D** The protein expression level of SOX10 in WT and KO cells was detected by western blotting. **E** The protein expression level of SOX10 in WT and KO cells was further confirmed by immunofluorescence assay. Scale bar, 50 μm. **F** Cell morphology was observed by phase-contrast microscope. Scale bar, 250 μm.

Single-guide RNAs targeting the human *SOX10* locus (exon 1 or exon 2; Fig. 1A) were designed using E-CRISP tools. Three pairs of annealed guide oligos were chosen and cloned into the CRISPR-Cas9 expression vector pSpCas9 (BB)-2A-GFP (PX458) (Addgene, Cambridge, MA, USA; #48138) by T4 DNA ligase (TaKaRa Bio, Kusatsu, Japan). To evaluate the targeting efficiency, three different plasmids were separately transfected into 293FT cells using Lipofectamine 2000 transfection reagent (Invitrogen). Two days after transfection, genomic DNA was extracted from transfected 293FT cells, and the targeting efficiency was verified by a T7EN1 assay (New England Biolabs, Ipswich, MA, USA) and genome sequencing. The most efficient sgRNA (sgRNA2) was selected for *SOX10* targeting in hiPSCs.

### Generation of SOX10-knockout (KO) hPSC lines using CRISPR-Cas9 technology

To generate SOX10-KO cell lines, hPSCs were treated with 10 mM Y27632 (Sigma-Aldrich, St. Louis, MO, USA) for 24 h. Then, 2 × 10^6^ hPSCs were dissociated by Accutase (Invitrogen) and resuspended in 10 μg plasmid containing Cas9 and sgRNA2 in 82 μl of P3 Nucleofector solution and 18 μl of supplement (Lonza, Gaithersburg, MD, USA). Electroporation was performed using an Amaxa 4D Nucleofector system with the CA137 program (Lonza). Transfected cells were seeded in Matrigel-coated petri dishes and cultured in mTesR1 medium with 10 µM Rock inhibitor (Y-27632) for the first 24 h. The next day, cells were dissociated, and GFP^+^ single cells were sorted into 96-well plates by fluorescence-activated cell sorting (FACS). A total of 20 isolated clones were expanded into 24-well plates and used for PCR screening. Successful knockout cell lines were verified by genome sequencing and western blot. Genomic DNA was extracted using a TIANamp Genomic DNA Kit (Tiangen Biotech, Beijing, China). PCR was performed using an Ultra HiFidelity PCR Kit (Tiangen Biotech) following the manufacturer’s instructions. PCR-amplified DNA was then purified using a QIAquick Gel Extraction Kit (QIAGEN, Valencia, CA, USA), and DNA sequences were confirmed by the Sangon DNA sequencing service. A list of potential off-target sites was generated by the online CRISPR-Cas9 prediction tool (Cas-OFFinder). Then, the top 10 potential off-target sites were chosen, PCR amplified and sequenced. Primers used for amplification of the genomic locus are displayed in Supplementary Table 1.

Two SOX10-KO cell lines, namely, *SOX10*^−/−^ hiPSCs 1 (KO1) and *SOX10*^−/−^ hiPSCs (KO2), were selected, and their pluripotency was detected by immunostaining for OCT4, SOX2, NANOG and TRA-1-60. The differentiation ability of SOX10-knockout hiPSCs to all three embryonic germ layers was assessed by immunostaining of the endodermal marker SOX17, the mesodermal marker αSMA, and the ectodermal marker TUBB3 in vitro and teratoma formation in vivo in 8 weeks old male SCID mice (Vital River Laboratories, Beijing, China). All experimental procedures involving animals were approved by the Animal Ethics Committee of Sun Yat-sen University.

### Neural crest differentiation of hPSCs

Neural crest induction was performed using monolayer culture protocols as previously described [30]. In brief, hiPSCs were dissociated into single cells with Accutase and then replated on Matrigel-coated dishes at a density of 20,000 cells/cm^2^. At the beginning of the induction (day 1), cells were incubated with E6 medium for 24 h and then cultured in NCN2 medium for 6 days (7 days in total). p75^high^HNK1^+^ NCSCs were isolated by fluorescence-activated cell sorting (FACS) using a BD Influx Cell Sorter (BD-Pharmingen), cultured and expanded in neural crest culture medium (NCCM).

In the migration assay, day 7 differentiated cells were disaggregated and suspended in neural crest induction medium (NIM) to form spheres in ultralow-attachment culture dishes for 1 day. Then, the spheres were attached to dishes coated with poly-L-ornithine (PO; Sigma-Aldrich) and laminin (LN; Millipore, Temecula, CA, USA) and cultured in NIM for 24–48 h.

### Peripheral neuron differentiation of NCSCs

For peripheral neuronal differentiation, NCSCs were plated onto dishes precoated with PO/LN and cultured in DMEM/F12 induction medium supplemented with N2 supplement (Gibco), 1x GlutaMAX (Gibco); glial cell line-derived neurotrophic factor (GDNF, 10 ng/ml), brain-derived neurotrophic factor (BDNF, 10 ng/ml), nerve growth factor (NGF, 10 ng/ml), neurotrophin-3 (NT3, 10 ng/ml) (all from PeproTech, Rocky Hill, New Jersey, USA); 200 mM ascorbic acid (Sigma-Aldrich); and 0.5 mM cAMP (Sigma-Aldrich). The medium was replaced every 3–4 days. After 3–4 weeks of culture in differentiation medium, cells were analyzed for the expression of peripheral neural markers such as Peripherin (PRPH) and β-III Tubulin (TUJ1) by immunocytochemistry and qRT-PCR.

### Schwann cell differentiation of NCSCs

For Schwann cell differentiation, NCSCs were expanded for more than 8 weeks in vitro and then cultured in knockout DMEM/F-12 medium supplemented with N2 supplement, 10 ng/mL ciliary neurotrophic factor (CNTF; PeproTech), 0.5 mM dibutyryl-cAMP (dbcAMP; Sigma-Aldrich) and 20 ng/mL neuregulin (NRG, Sigma-Aldrich) for 3–4 weeks. The medium was refreshed every 3–4 days. The resulting cells were assessed by immunostaining and qRT-PCR with the Schwann cell markers GFAP and S100B.

### Cell migration and imaging

The NCSCs were seeded at a density of 1 × 10^4^ cells/cm^2^ at 37 °C with 5% CO_2_ in an enclosed chamber. The migration ability of cells was monitored for 24 h and detected by a BioTek Lionheart FX Automated Live Cell Imager (BioTek Instruments, Inc., Winooski, VT, USA).

### RNA extraction and qRT-PCR

Total RNA was extracted from cells with TRIzol Reagent (Invitrogen) and treated with DNase I for 30 min at 37 °C to avoid genomic contamination. For RT-PCR, 1 μg of RNA was reverse transcribed into cDNA using a First Strand cDNA Synthesis Kit (Thermo Fisher Scientific, Rutherford, NJ, USA) following the manufacturer’s instructions. qRT-PCR was performed on a LightCycler 480 Detection System (Roche Diagnostics, Branchburg, NJ, USA) using a DyNAmo HS SYBR Green qPCR Kit (Thermo Fisher Scientific). The relative quantification of gene expression was carried out according to the 2^−ΔΔCt^ method. Housekeeping gene for standardizations was *β-actin* (*ACTB*). Primer sequences are listed in Supplementary Table 2.

### Western blotting

Cells were homogenized with RIPA lysis buffer containing a protease inhibitor mixture (Thermo Fisher Scientific). The homogenates were centrifuged at 15,000 × *g* at 4 °C for 10 min, and the supernatants were collected. Approximately 20 μg of total protein extract was subjected to 10% SDS-PAGE gels and then transferred onto polyvinylidene fluoride membranes (Millipore, Temecula, CA, USA). The membranes were blocked with 1% BSA in TBS for 1 h and then probed with anti-SOX10 antibody (Abcam) or other primary antibodies (Abcam) at 4 °C overnight, followed by reaction with horseradish peroxidase (HRP)-linked secondary antibodies (Cell Signaling Technology, Beverly, MA, USA) at room temperature for 1 h. The membranes were then imaged on the ChemiDoc™ Touch Imaging System (Bio-Rad Laboratories, Inc., Hercules, CA, USA). Information of the antibodies is listed in Supplementary Table 3.

### Flow cytometry analysis

NCSCs from control hiPSCs and SOX10-KO hiPSCs were isolated or examined by FACS. Cells were dissociated with Accutase into single-cell suspensions and incubated with monoclonal antibodies against human antigens, including p75, HNK1, or CD49D (all from BD Biosciences). An irrelevant isotype-identical antibody (BD Biosciences) served as a negative control. Data were analyzed using CytExpert software 2.0 (Beckman Coulter, CA, USA). Information of the antibodies is listed in Supplementary Table 4.

### Immunofluorescence staining

Cells were fixed with 4% paraformaldehyde, washed three times in 1× PBS, and then permeabilized with 0.3% Triton X-100 (Sigma) for 30 min, followed by incubation with primary antibodies at 4 °C overnight. After washing with PBS three times, the cells were incubated with a secondary antibody conjugated to Alexa Fluor 488 or Alexa Fluor 555 at room temperature for 1 h in the dark. The nuclei were counterstained with 4′, 6-diamidino-2-phenylindole (DAPI; Sigma-Aldrich). Images were analyzed by using a confocal microscope. The primary and secondary antibodies used are listed in Supplementary Table 5.

### Apoptosis assays

For flow cytometry-based detection, apoptotic cells were measured using an Annexin V-FITC/PI Apoptosis Detection Kit (BD Biosciences) according to the manufacturer’s instructions. Briefly, cells were harvested and washed twice with PBS, resuspended in binding buffer with Annexin V-FITC and PI for 15 min, and then analyzed by a flow cytometry assay. Annexin V-positive cells were considered apoptotic cells.

TUNEL staining was performed by using a fluorescein in situ cell death detection kit to analyze the distribution of cells undergoing apoptosis (Roche) following the manufacturer’s instructions. Samples were counterstained with DAPI and then directly analyzed with a fluorescence microscope.

### Cellular reactive oxygen species (ROS) assay

Here, acetic 5-(chloromethyl)-2-(3, 6-diacetoxy-2,7-dichloro-9H-xanthen-9-yl) benzoic anhydride (CM-H2DCFDA; Thermo Fisher Scientific) was used as a fluorescent indicator for reactive oxygen species (ROS) detected by FACS and fluorescence microscopy. For FACS analysis, cells were trypsinized, washed twice with PBS, centrifuged at 200 × *g* for 5 min, and suspended in 500 µL of PBS. The cells were then incubated with 20 µM CM-H2DCFDA for 30 min at 37 °C and detected by flow cytometry. For fluorescence microscopy, cells were washed with PBS, fixed with 70% methanol for 10 min, and stained with 20 µM DCFDA for 30 min at 37 °C. Then, the cells were examined under a Zeiss LSM800 confocal microscope (Oberkochen, Germany).

### Drug treatment

We test whether the ROS scavenger, N-acetylcysteine (NAC) [31], could protect the NCSCs from ROS-mediated cell death. NAC (Sigma-Aldrich) was dissolved in deionized water as stock solutions of 1 mM (pH 7.0) and added directly to complete cell culture media with a final concentration of 5 mM for 24 h at 37 °C. Then, treated cells were washed with PBS, trypsinized, resuspended in PBS. ROS levels were detected by CM-H2DCFDA assay and cell apoptosis was evaluated using the Annexin V-FITC Kit as described above.

### Cell viability and Cell proliferation analysis

Cell viability was measured by cell count with trypan blue dye (Sigma-Aldrich) using Automated Cell Counters (Nexcelom, Massachusetts, USA) at indicated time points during neural crest differentiation. For cell proliferation assay, neural crest spheres formed by day 7 differentiated cells were dissociated. Then 5 × 10^3^ cells were seeded to a 96-well plate and cultured for 0, 24, and 48 h. Then, 100 μl of Cell Counting Kit-8 (CCK-8) reagent (Dojindo, Kumamoto, Japan) was added to each well for 2 h incubation at 37 °C. The absorbance was measured at 450 nm using Infinite 200 PRO multimode plate reader (Tecan, Switzerland).

### Chromosomal microarray analysis (CMA)

Chromosomal microarray analysis (CMA) was used to measure copy number variants (CNVs) and analyze gains and losses of DNA throughout the genome of control hiPSCs (WT1) and two SOX10 knockout hiPSC lines (KO1 and KO2) by the CytoScan 750 K array (Affymetrix; Themo Fisher Scientific). CMA could detect human genomic DNA CNVs and loss of heterozygosity with ≥50 probe labels and ≥200 kb resolution, covering 22 pairs of autosomal and sex chromosomes. The thresholds for screening were set at ≥200 kb for gains (duplications), ≥200 kb for losses (deletions).

### RNA sequencing

Genome-wide transcriptional profiles of day 7 neural crest cells derived from control hiPSCs and SOX10-knockout (KO1- and KO2-) hiPSCs, including WT1-NC (2 samples), KO1-NC (2 samples) and KO2-NC (2 samples), were identified by RNA sequencing. The RNA libraries were prepared from the total RNA and constructed using an Illumina mRNA-seq Prep Kit (Illumina, San Diego, CA, USA). The fragmented and randomly primed 150 bp paired-end libraries were sequenced using Illumina HiSeq 2000. Sequencing data were processed using Consensus Assessment of Sequence and Variation (CASAVA, version 1.8.2; Illumina) using the default settings. The reads per kilobase of transcript per million mapped reads (RPKM) value of each gene was calculated and normalized using Cufflinks. Pearson’s correlation coefficients were calculated (R^2^) to measure the similarities of the global gene expression profiles between neural crest cells from different cell lines. The RNA-Seq data were also analyzed using Ingenuity Pathways Analysis (IPA) software (Ingenuity Systems, Inc., Redwood City, CA, USA) to categorize the differentially regulated genes. RNA-seq data have been deposited in the Gene Expression Omnibus (GEO) under the accession number GSE160312.

### Statistical analysis

All experiments shown were biologically replicated for at least three times. All results were shown as mean ± SD from at least three independent experiments. GraphPad Prism 7 Software was used for statistical analysis. ImageJ 1.52a software was used to analyze the contractile experiment and immunostaining images. CytExpert 2.0 and Flow Jo V 10.0 were used to analyze the FACS data. Ingenuity Pathways Analysis (IPA; Version: 52912811) software (Ingenuity Systems, Inc.) was used for analyzing the RNA-Seq data. The significance of the difference between the mean values was determined by one-way analysis of variance (ANOVA). Differences were considered significant when *p* < 0.05.

## Results

### Generation and characterization of SOX10-KO hiPSCs

To generate the SOX10-deficient hiPSC model, we designed single-guide RNAs targeting exon 1 or exon 2 of the *SOX10* locus, which is close to or within the HMG domain (Fig. 1A). The targeting efficiency was analyzed by T7EN1 assay and Sanger sequencing (Fig. 1B). The guide RNA (sgRNA2) with the highest targeting efficiency was selected for SOX10-knockout in hiPSCs.

HEF-hiPSCs were transfected with the PX458 vector containing sgRNA2, Cas9, and GFP cassettes by electroporation. Single GFP^+^ cells were sorted by FACS, replated onto Matrigel-coated 96-well plates (1 cell/well), and expanded for Sanger sequencing. Two colonies containing different homozygous alleles with frame-shifted coding sequences, which may result in premature stop codons, were obtained (referred to as KO1 and KO2; Fig. 1C). Then mutated hiPSC lines and control hPSCs (wild-type hiPSCs and H9 embryonic stem cells; designated WT1 and WT2) were induced to differentiate spontaneously in serum-free medium. The western blotting revealed that abundant SOX10 protein was expressed in differentiated cells of the control group, while the SOX10 protein in mutant cells could hardly be detected (Fig. 1D). These results were consistent with those of the immunofluorescence assay (Fig. 1E). To verify whether off-target mutations exist in these cell lines, the top 10 potential off-target sites were analyzed, and no mutation was detected by Sanger sequencing in these sites (Supplementary Fig. S1A). To further detect whether CRISPR/Cas9 gene editing could lead to diverse type of genetic alterations, we performed chromosomal microarray analysis (CMA) to identify structural chromosomal aberrations in SOX10-KO hiPSCs. The results illustrated that no aneuploidy or structural rearrangement was found in either parental hiPSCs or SOX10-KO hiPSCs. Moreover, no extra deletions (red triangle) or duplications (blue triangle) on chromosomes were detected in SOX10-KO group compared to control hiPSCs (Supplementary Fig. S1B). The above evidence demonstrates that the SOX10-knockout cell lines were successfully generated. The KO1 and KO2 hiPSCs could be maintained with typical cell morphology and a normal proliferation rate during long-term in vitro culture (Fig. 1F), and highly expressed pluripotency markers (Supplementary Fig. S2A). In addition, we found that SOX10-knockout cells could differentiate into cells or tissues of all three germ layers in vitro and in vivo (Supplementary Fig. S2B, C). These data suggest that SOX10 knockout did not influence the pluripotency of hiPSCs.

### SOX10 deficiency caused migration defects during neural crest cell differentiation of hiPSCs

**Fig. 2:**
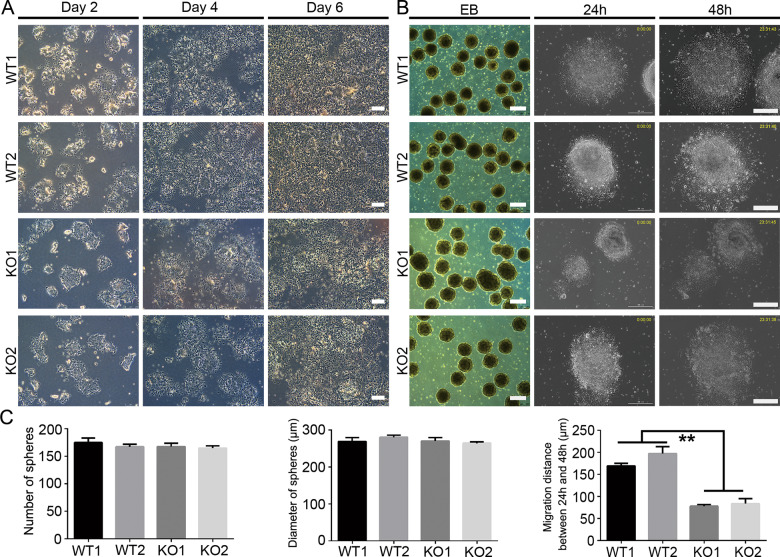
Nueral crest differentiation of SOX10-knockout hiPSCs. **A** The morphology change during neural crest differentiation from WT and SOX10-knockout cell lines was detected under phase-contrast microscopy. Scale bar, 250 μm. **B** Sphere formation and attachment for detection of migration ability in day 7 differentiated WT and KO cells. Scale bar, 300 μm. **C** The number of spheres, diameter of spheres, and migration distance between 24 h and 48 h after attachment was calculated and compared. *n* = 3 biological replicates. The results are the mean ± SD. **p < 0.01.

To induce NCSC differentiation from SOX10-KO hiPSCs, we used a 7-day monolayer protocol as described [30] that mimicked normal neural crest development. Two days after induction, WT and KO cells proliferated and became cluster-like small colonies, and no obvious difference in the cell/colony morphology was detected (Fig. 2A). After 4 days of differentiation, WT cells seemed to migrate or proliferate more quickly compared to cells in the KO group (Fig. 2A). Moreover, significant morphological changes occurred, and a large number of multipolar cells with narrow projections (neural crest-like cells) emerged in the WT group. However, these cells rarely appeared in KO group. Six days later, we also noticed that compared to the cultures of the WT group, the cultures of the KO group showed a reduced cell density or cell number (Fig. 2A).

We then tried to determine whether loss of SOX10 may cause defects in proliferation during neural crest induction of hiPSCs. Cell numbers were counted and the results indicated that the cell density in KO1 and KO2 groups were similar to that in WT group at different time points (day 2, day 4 and day 6; Supplementary Fig. S3A). Anti-PCNA immunostaining showed that the number of PCNA^+^ cells in the KO group was similar to that in the WT group on day 7 (Supplementary Fig. S3B). To determine whether SOX10 deletion affects the migration of differentiated cells, day 7 cells were collected and cultured in suspension to form spheres for 1 day. We found that the numbers and diameters of spheres were not significantly different between the WT and KO groups (Fig. 2B, C). Then, the spheres were replated onto PO/LN-coated plates and cultured for 48 h. Cells continuously migrated out from the spheres, and we found that the cell migration distance between 24 h and 48 h in the KO group (KO1: 77.8 ± 3.974 μm; KO2: 83.9 ± 11.15 μm) was drastically lower than that in the WT group (WT1: 169.2 ± 6.03 μm; WT2: 197.5 ± 15.28 μm) (*p* < 0.01; Fig. 2B, C). CCK8 assay further showed that no statistically significant difference in proliferation ability of the sphere cells was observed between WT and KO groups (Supplementary Fig. S3C). The above data show that SOX10 knockout impaired the cell migration ability during neural crest cell differentiation of hiPSCs.

### Development of the postmigratory neural crest is severely disrupted in SOX10-deficient hiPSCs

**Fig. 3:**
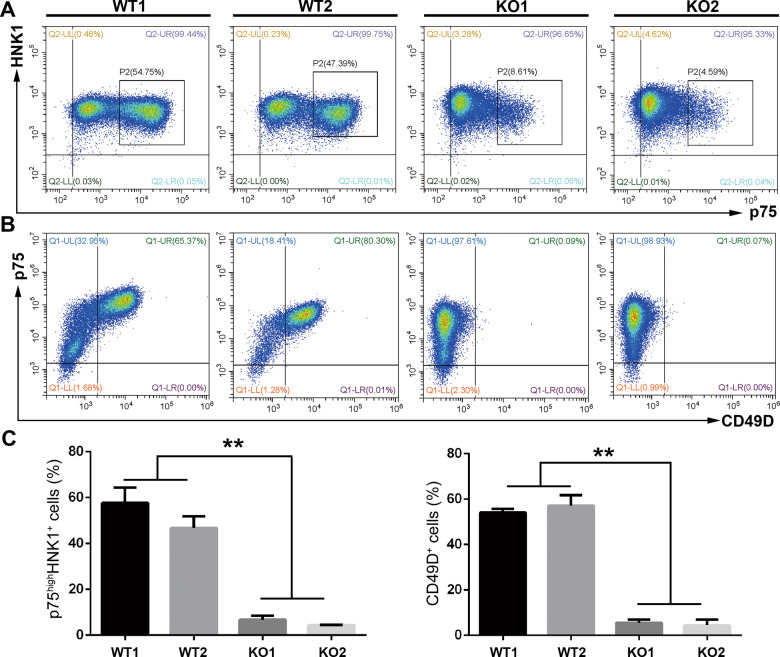
FACS analysis of migrating neural crest stem cells derived from WT and KO hPSCs. **A** The proportion of p75^high^/HNK1^+^ migrating NCSCs in day 7 differentiated cells of WT and KO groups was analyzed by FACS. **B** The proportion of p75^high^/CD49D^+^ migrating NCSCs in day 7 differentiated cells of WT and KO groups was analyzed by FACS. **C** The proportion of p75^high^/HNK1^+^ and p75^high^/CD49D^+^ in day 7 differentiated cells from different groups were calculated and compared, respectively. *n* = 5 biological replicates. Columns represent the mean ± SD. **p < 0.01.

**Fig. 4:**
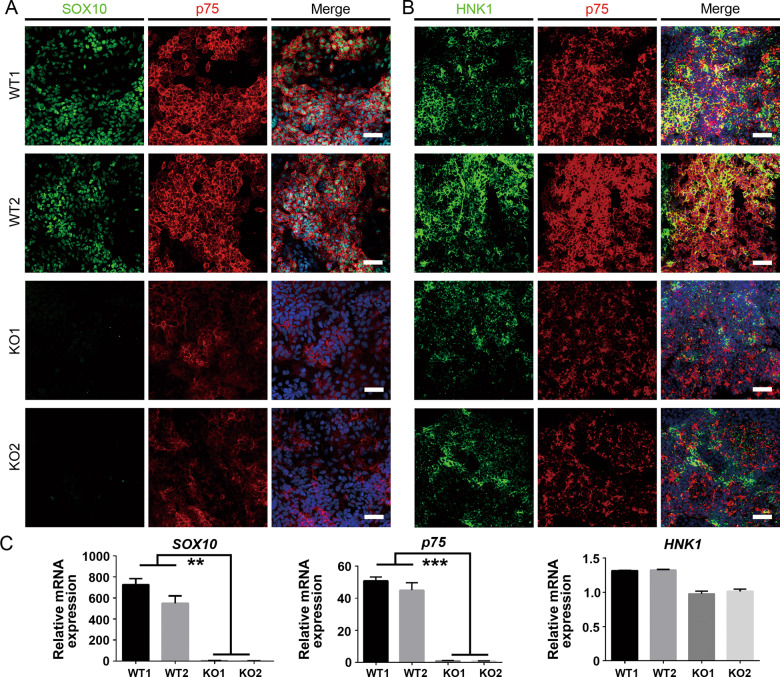
Characterization of day 7 differentiated cells in WT and KO groups. **A** Expression of migrating neural crest markers SOX10/p75 in day 7 differentiated cells in WT and KO groups were detected by immunostaining. Scale bar, 50 μm. **B** Expression of migrating neural crest markers p75/HNK1 in day 7 differentiated cells in WT and KO groups were detected by immunostaining. Scale bar, 50 μm. **C** The mRNA level of migrating neural crest markers (*SOX10*, *p75*, *HNK1*) in day 7 differentiated cells was detected by qRT-PCR. *n* = 3 biological replicates. Columns represent the mean ± SD. **p < 0.01, ***p < 0.001.

It was reported that HNK1^+^/p75^high^ or CD49D^+^ cells were truly migrating NCSCs [32, 33]. Therefore, day 7 cell population was analyzed by FACS. We discovered that the percentage of HNK1^+^ cells in the KO group (≥97%) was similar to that in the control group (≥98%), and most HNK1^+^ cells co-expressed p75. However, there was a significantly lower number of HNK1^+^/p75^high^ NCSCs in the SOX10-KO group (KO1: 6.79 ± 1.8%; KO2: 4.337 ± 0.255%) than in the WT group (WT1: 57.68 ± 6.747%; WT2: 46.75 ± 5.096%; *p* < 0.01) (Fig. 3A). Interestingly, CD49D was expressed by more than 50% of cells in the WT group, while only a small population of CD49D^+^ cells could be detected in the SOX10-KO group (KO1: 5.513 ± 1.476%; KO2: 4.42 ± 2.447%; *p* < 0.01) (Fig. 3B, C). Moreover, immunofluorescence assays revealed that most of the day 7 cells in the control group co-expressed p75/HNK1 in the plasma membrane and SOX10 in the cell nucleus (Fig. 4A, B). However, SOX10^+^ cells were not detected in the KO group. Although most of the KO cells still expressed p75 and HNK1, the number of p75^high^ cells in the KO group was greatly reduced compared with that in the WT group. qRT-PCR analysis further demonstrated that differentiated WT cells highly expressed postmigratory markers *p75* and *SOX10*, while significantly lower mRNA transcript levels of these markers were detected in SOX10-KO cells. Interestingly, there was no statistically significant difference in the mRNA level of *HNK1* between the control and KO groups (Fig. 4C). These results indicate that ablation of SOX10 markedly impaired the generation of postmigratory neural crest cells from hiPSCs.

**Fig. 5:**
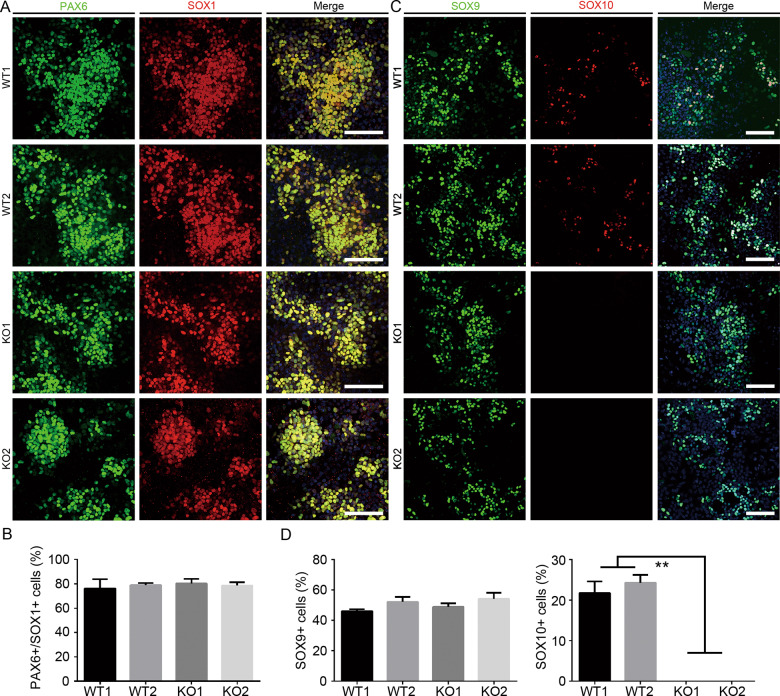
Characterization of day-3 and day-5 differentiated cells in WT and KO groups. **A** Expression of neural epithelial cell markers PAX6 and SOX1 in day-3 differentiated cells in WT and KO groups was detected by immunostaining. Scale bar, 100 μm. **B** The number of PAX6^+^/SOX1^+^ cells in day-3 differentiated cells was calculated and compared. **C** Expression of neural epithelial cell markers SOX10 and SOX9 in day-5 differentiated cells in WT and KO groups was detected by immunostaining. Scale bar, 100 μm. **D** The number of SOX10^+^ and SOX9^+^ cells in day-5 differentiated cells was calculated and compared, respectively. *n* = 5 biological replicates. Columns represent the mean ± SD. **p < 0.01.

To explain how SOX10 deletion impairs postmigratory neural crest cell commitment, we tried to determine whether loss of SOX10 inhibits the neural conversion of hiPSCs. The results of immunostaining exhibited a similar percentage of SOX1^+^ and PAX6^+^ cells in both groups on either day 3 (~80%; Fig. 5A, B) or day 7 (<5%; Fig. S4A). NESTIN, a marker of both neural stem cells and NCSCs, was expressed abundantly in most WT and KO cells on day 7 (Supplementary Fig. S4A). The qRT-PCR results also showed that the mRNA levels of these neural stem cell markers in the KO group were not significantly different from those in the WT group (Supplementary Fig. S4B). These preliminary data indicate that *SOX10* is not involved in the neural conversion of hiPSCs.

**Fig. 6:**
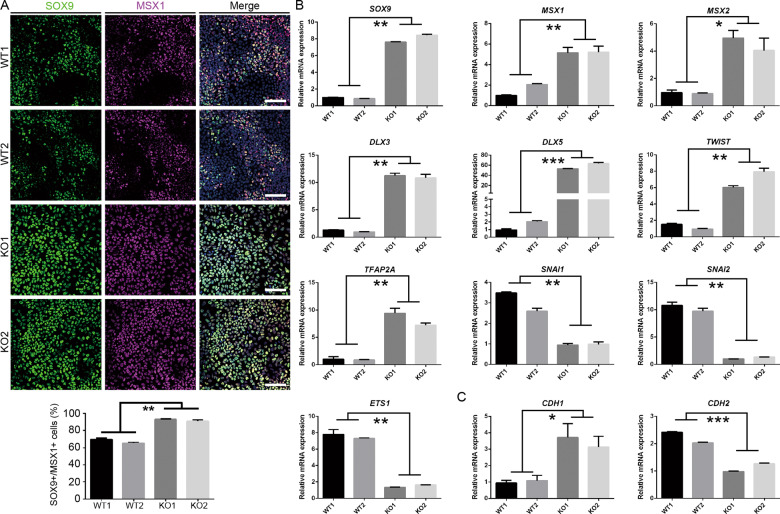
Detection of the expression premigratory and postmigratory neural crest markers in day 7 differentiated cells. **A** SOX9^+^/MSX1^+^ premigratory neural crest stem cells in day 7 differentiated cells were detected, counted and compared. Scale bar, 100 μm. **B** qRT-PCR analysis for the mRNA expression of premigratory and postmigratory neural crest markers in day 7 differentiated cells in different cell lines. **C** qRT-PCR analysis for the mRNA expression of cadherin family members *CDH1* and *CDH2*. *n* = 3 biological replicates. Columns represent the mean ± SD. *p < 0.05, **p < 0.01, ***p < 0.001.

We then asked whether SOX10 knockout could block neural crest cell commitment from neural epithelial cells. Previous studies reported that BMP, WNT, and FGF signaling pathways work coordinately to activate the expression of premigratory neural crest-specific genes (*MSX1*, *MSX2*, *DLX5*, *SOX9*, *TWIST*, etc.) in neural epithelial cells of the dorsal neural tube. Then premigratory neural crest cells subsequently undergo epithelial-to-mesenchymal transition (EMT) and become migrating NC cells, which highly express migration-related genes including *p75*, *SOX10*, *SNAI1*, *SNAI2*, *ETS1*, and *CDH2* [34, 35]. Therefore, we detected the expression of typical premigratory markers by immunofluorescence assay and found that comparable SOX9^+^ premigratory NC cells were generated on day 5 of differentiation in all the cell lines tested. We also noted that ~20–30% of the SOX9^+^ cells also expressed SOX10 in the WT group on day 5 (Fig. 5C, D). However, more SOX9^+^/MSX1^+^ premigratory cells could be found in day 7 KO group cultures (KO1: 93.01 ± 0.01%; KO2: 91.05 ± 0.13%) than in WT group (WT1: 69.38 ± 0.02%; WT2: 65.25 ± 0.01%; Fig. 6A). qRT-PCR further confirmed that the mRNA expression levels of premigratory markers, including *MSX1*, *MSX2*, *DLX3*, *DLX5*, *SOX9*, *TFAP2A*, and *TWIST*, were strikingly upregulated on KO cells compared with those on WT cells (Fig. 6B).

More importantly, we revealed that the mRNA levels of migratory NC markers such as *SNAI1*, *SNAI2*, and *ETS1* in KO cells were notably lower in the KO group than in the WT group (Fig. 6B). It has been reported that the cadherin switch, due to the loss of E-cadherin (CDH1) expression and the concomitant upregulation of N-cadherin (CDH2) expression, greatly attenuates the adhesiveness of NC cells and significantly increases their motility [35]. Our qRT-PCR results showed that the mRNA level of *CDH1* in KO cells was ~3–4-fold higher than that in WT cells, while the *CDH2* mRNA level in the KO group was remarkably reduced compared to that in the WT group (Fig. 6C). These data suggest that SOX10 deficiency could significantly inhibit the transition of migratory NC cells from premigratory NC progenitors rather than affect neural crest cell commitment from neural epithelial cells.

Previous studies showed that SOX10 has a different enhancer responsible for cranial than for trunk expression [36]. We then tried to determine which regional type of neural crest cells were generated in our study. The qRT-PCR analysis displayed that markers for cranial NC (*LHX5*, *OTX2*) were sharply increased, while vagal- (*HOXB3*, *HOXB5*), trunk- (*HOXC8*, *HOXC9*), and sacral- (*HOXD12*, *HOXD13*) specific genes remained largely unchanged on day 7 differentiated cells when compared to day 0 undifferentiated hiPSCs (Supplementary Fig. S5A). These results indicated that cranial NCSCs were successfully derived from hiPSCs in our differentiation system. Moreover, to discover whether SOX10-knockout would affect the trunk specification of hiPSCs, cells were induced to differentiate to trunk neural crest as previously described [37]. We found that the percentage of p75^high^HNK1^+^ postmigratory NCSCs in KO groups was substantially decreased compared to that in WT group as revealed by FACS analysis (Supplementary Fig. S5B, C), although the differentiated cells in both WT and KO groups highly expressed trunk markers *HOXC8* and *HOXC9* (Supplementary Fig. S5D). The qRT-PCR analysis also revealed that the mRNA transcripts of postmigratory markers including *SOX10*, *SNAI1*, and *SNAI2* were significantly lower in KO cells than WT group (Supplementary Fig. S5E). The above evidence demonstrates that SOX10 deletion considerably impairs postmigratory trunk neural crest specification from hiPSCs.

**Fig. 7:**
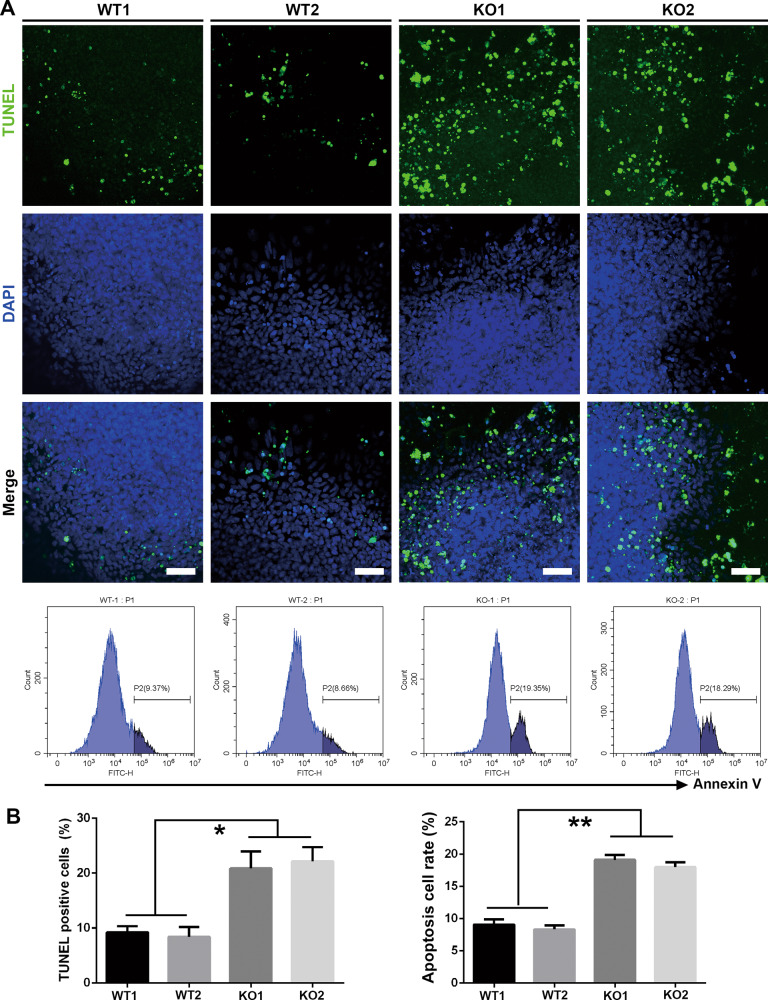
Detection of the cell apoptosis in day 7 differentiated cells. **A** The cell apoptosis in day 7 cell cultures in different groups was analyzed by TUNEL assay and Annexin V staining. Scale bar, 50 μm. **B** The TUNEL^+^ and Annexin V^+^ cell ratio was calculated and compared, respectively. *n* = 3 per group. Columns represent the mean ± SD. *p < 0.05, **p < 0.01.

In mice, increased cell apoptosis was detected in vagal and trunk neural crests derived from both homozygous SOX10 mutation and knockout model mice [21, 38, 39]. In our study, we found that the number of TUNEL-positive cells in the KO group (KO1: 20.89 ± 3.072%; KO2: 22.16 ± 2.612%) was considerably increased compared with that in the WT group on day 7 (WT1: 9.24 ± 1.129%; WT2: 8.403 ± 1.816%; *p* < 0.05; Fig. 7A, B). Similar results were obtained by Annexin V staining and subsequent FACS analysis (*p* < 0.01; Fig. 7A, B). Furthermore, since reactive oxygen species (ROS) play an important role in apoptosis induction [40], we tried to determine whether ROS stress is implicated in SOX10-KO-related cell death. The results of CM-H2DCFDA staining of day 7 differentiated cells showed that an increase in the production of ROS could be detected in the KO group (2~3-fold higher than that in the WT group), as quantified by relative fluorescence intensity using FACS (*p* < 0.05) (Supplementary Fig. S6A–C). We then added NAC, an ROS scavenger, to the differentiated cell cultures and discovered that treated with NAC significantly downregulated the ROS accumulation accompanied with remarkably lower amount of TUNEL^+^ apoptotic cells in KO group (Supplementary Fig. S6D, E), which indicate that NAC treatment could efficiently protect the NCSCs from ROS-mediated cell death. These data indicate that loss of SOX10 could impair the cell survival of human neural crest cell lineages, which may be partially due to the accumulation of ROS.

### Reduced neuronal and glial differentiation potential in SOX10-KO NCSCs

**Fig. 8:**
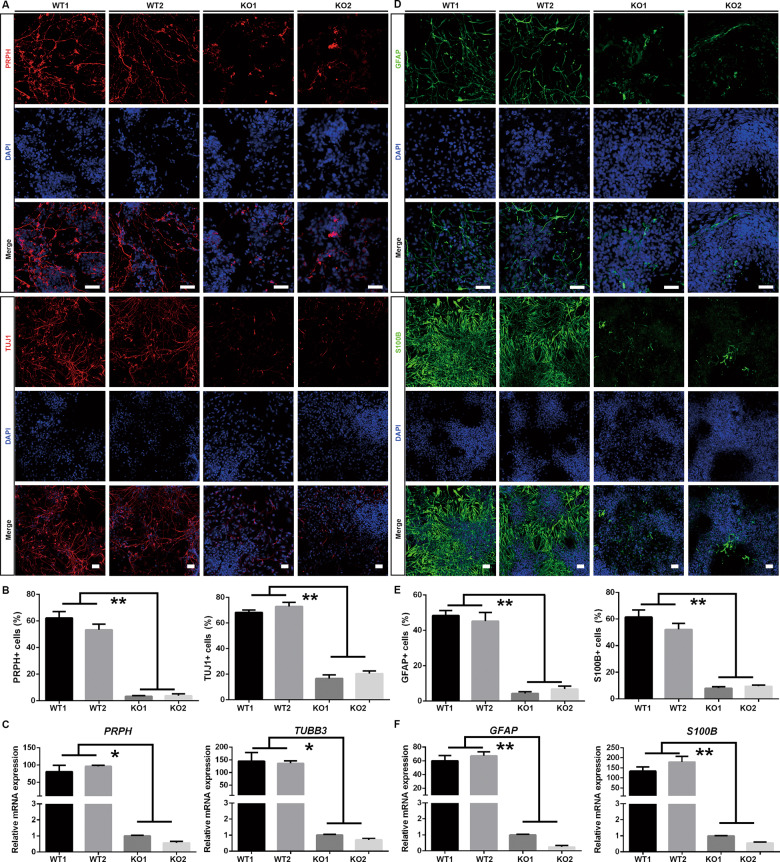
Neuronal and Schwann cell differentiation of NCSCs derived from WT and KO hiPSCs. **A** PRPH^+^ and TUJ1^+^ neurons were detected by immunofluorescence. Scale bar, 50 μm. **B** The ratio of PRPH^+^ and TUJ1^+^ neurons were counted and compared. **C** qRT-PCR analysis for the mRNA expression of *PRPH* and *TUJ1*. **D** GFAP^+^ and S100B^+^ Schwann cells were detected by immunofluorescence. Scale bar, 50 μm. **E** The ratio of GFAP^+^ and S100B^+^ Schwann cells were counted and compared. **F** qRT-PCR analysis for the mRNA expression of *GFAP* and *S100B. n* = 3 biological replicates. Columns represent the mean ± SD. *p < 0.05, **p < 0.01.

Control and SOX10-KO p75^high^/HNK1^+^ NCSCs were enriched by FACS and expanded in NCCM. No statistically significant difference in sphere diameter, the proportion of ki67^+^ cells, or cell apoptosis rate was noted in NCSCs of either group (Supplementary Fig. S7A–C). For peripheral neuronal differentiation, cells in both groups proliferated with a similar trend, and no obvious cell death was observed. After 3–4 weeks of induction, markedly fewer cells in the KO group displayed neuronal morphology and expressed the peripheral neuronal markers PRPH and TUJ1 than those in the WT group (PRPH: <5% in the KO group and >50% in the control group, *p* < 0.01; TUJ1: about 20% in the KO group and >60% in the control group, *p* < 0.01) (Fig. 8A, B). The result of qRT-PCR analyses was consistent with that of immunostaining assay (Fig. 8C). For Schwann cell differentiation, cells were well maintained in induction medium and minimal cell death was noted in both groups. The immunostaining assay showed that GFAP and S100B were strongly expressed in the control group (for GFAP, WT1: 48.37 ± 2.801%, WT2: 45.23 ± 4.819%; for S100B, WT1: 61.5 ± 5.274%, WT2: 52.23 ± 4.508%), while only a small number of GFAP^+^ and S100B^+^ cells could be detected in the KO group (for GFAP, KO1: 4.3 ± 0.9539%, KO2: 6.8 ± 1.735%, *p* < 0.01; for S100B, KO1: 8.033 ± 0.9866%; KO2: 9.467 ± 0.7506%, *p* < 0.01) (Fig. 8D, E). These results were consistent with the *GFAP* and *S100B* mRNA level as assessed by qRT-PCR (Fig. 8F). The above evidence demonstrated that SOX10 plays essential roles in the neuronal and glial differentiation ability of NCSCs.

### Global gene expression profiling of the neural crest from the WT and KO groups

To further characterize the properties of day 7 SOX10-KO neural crest cells, we performed a genome-wide transcriptional profile analysis of NC cells in both groups (GSE160312). We calculated the coefficients of determination (R2) for all expressed genes and revealed an extremely high level of similarity in the gene expression profile between 2 samples from independent cell lines respectively (R2å 0.94; Supplementary Fig. S8A), which suggests the excellent specificity and reproducibility of our neural crest differentiation protocol. However, lower similarity was detected in neural crest cells between WT and KO populations (R2 <0.90), which was consistent with the results of Principal component analysis (PCA) (Supplementary Fig. S8B). We further found that genes characteristic of putative neural epithelial cells (*SOX1*, *PAX6*, and others) were expressed at similarly low levels in both groups, while markers expressed by both neural stem cells and the neural crest cells (*SOX2*, *NESTIN*, and others) were enriched in WT and KO cells. More importantly, premigratory NC markers (*MSX1/2*, *DLX3/5*, and others) were highly expressed, while migratory NC markers such as *SOX10*, *SNAI1*, and others in the KO group were significantly downregulated compared with those in the WT group (Supplementary Fig. S8C). In addition, the result revealed that the expression of SOX10 target genes including *PMP22* and *MPZ* in KO group were remarkably lower than that in WT cells [41], which was consistent with the results of western blotting (Supplementary Fig. S8D). Analysis using Ingenuity Pathways Analysis (IPA) software (the selected set of genes with RPKM value ≥ 5) indicated that cell survival, migration, and neural development were greatly inhibited, while the levels of apoptosis and ROS generation in the KO group were considerably increased compared with those in the WT group (Supplementary Fig. S8E, F). Western blotting analysis further demonstrated that the protein level of NOXA1 and ROMO1 (ROS pathway members) dramatically increased in KO group when compared to WT group (Supplementary Fig. S8G). These RNA-Seq findings are in accordance with our above experimental results.

Intriguingly, we found that the mRNA transcripts of *TFAP2C*, a predominant transcription factor of nonneural ectoderm (NNE), were 2-3-fold higher in SOX10-KO cells than in wild-type cells. We then analyzed the expression of TFAP2C in day 7 differentiated cells. The results of immunostaining showed that the percentage of TFAP2C^+^ cells was much higher in the KO group (KO1: 17.37 ± 2.201%; KO2: 21.67 ± 1.332%) than in the WT1 group (WT1: 7.2 ± 0.8888%; WT2: 6.4 ± 0.8%) (Supplementary Fig. S9A, B). qRT-PCR also confirmed that the mRNA levels of NNE markers, including *TFAP2C* and *KRT16*, in KO cells were significantly upregulated compared with those in WT cells (Supplementary Fig. S9C). These preliminary data indicate that SOX10 knockout may lead to a differentiation switch from neural crest to NNE cells.

## Discussion

In this study, we successfully established SOX10-knockout hiPSCs and discovered that SOX10 deletion impaired neural crest survival and inhibited the generation of migratory neural crest from premigratory progenitors, as illustrated by the higher numbers of premigratory NCSCs and lower numbers of migrating NCSCs, decreased migration ability, upregulated apoptosis rate, and compromised multipotency in the SOX10-KO group compared to the WT group. Moreover, we found that SOX10-knockout hESCs also possessed similar phenotypes including postmigratory neural crest differentiation defects, and increased cell apoptosis rate and ROS production with SOX10-knockout hiPSCs during neural crest differentiation (Supplementary Fig. S10).

The cellular function of SOX10 in NC development was extensively studied using several mutant animal models and showed that SOX10 ablation did not affect the induction, formation and emigration of neural crest cells, and normal numbers of premigratory NC could be detected in *SOX10* mutant mouse and zebrafish models [18, 20]. Here, we used hPSCs as an in vitro model for neural crest cell commitment. Our results showed that PAX6^+^ and SOX1^+^ neural epithelial cells (on day 3) and SOX9^+^ premigratory NCSCs (on day 5) can be efficiently generated from both WT and KO hiPSCs, which indicates that *SOX10* is not required for the commitment of neural epithelial cells or premigratory NCSCs from hiPSCs. These results were consistent with those of animal studies. Then, these premigratory progenitors underwent EMT, displayed extensive migration, and became SOX10^+^ migrating NCSCs in the WT group. However, the migration capability of the SOX10-deficient NCSCs was greatly restrained and p75^high^/HNK1^+^ or CD49D^+^ migrating cells could hardly be detected in SOX10-null cells, which indicates that SOX10 may play an essential role in the transition of postmigratory cells from premigratory NCSCs.

Although SOX10 is conservatively expressed in NCSCs in vertebrates, its expression pattern in different model organisms may differ. Previous studies showed that SOX10 was enriched in both premigratory and postmigratory NCSCs in *Xenopus laevis* and zebrafish [20, 42]. While in mice and chicks, SOX10 was primarily expressed by migrating NCSCs [43, 44]. Studies using human embryos revealed that SOX10 appeared in only the postmigratory cell population, showing a similar expression pattern to that of mice [45, 46]. This evidence suggests that SOX10 may have diverse roles in NC development in different species. In this study, we found that *SOX10* was mainly expressed by day 7 migrating NCSCs, which was in line with the results from human embryo studies. Interestingly, a small number of day 5 SOX9^+^ cells coexpressed SOX10 in the WT group were detected, and we found that these cells also expressed migration-related gene *SNAI2* by immunofluorescence staining assay (Supplementary Fig. S11). These preliminary data indicate that these SOX10^+^ cells may belong to the intermediate stage between premigratory and postmigratory NC cells.

Previous studies showed that SOX10 may serve as an important survival factor in multipotent NCSCs, yet the underlying mechanism remains largely elusive. Here, we did observe that the apoptosis rate in SOX10-KO group was increased versus that in the WT group. Furthermore, we found that ROS accumulation in SOX10-null cells was dramatically higher than that in WT cells as displayed by CM-H2DCFDA staining and RNA-Seq. These data suggest that SOX10 may be involved in the regulation of ROS generation or disposal, and the increased cell death of *SOX10*^−/−^ cells may be partially attributed to the accumulation of ROS. SOX10 was also implicated in the proliferation of neural crest cells [47], thus helping to maintain the neural crest cell progenitor pool. Therefore, researchers postulated that apoptosis of *SOX10* mutant cells may be a secondary consequence of the self-renewal and lineage commitment failures [18, 48]. In our study, however, we did not observe a decreased proliferation capacity in NCSCs of the KO group. These findings indicate that SOX10 may not be involved in the self-renewal of human neural crest cells.

SOX10 was shown to be required for the development of various neuronal and glial derivatives [18]. It was reported that the enteric neural crest of heterozygous SOX10^Lacz/+^or SOX10^Dom/+^steadily lost its progenitor state and differentiated into enteric neuroblasts [21, 49]. In addition, Sonnenberg-Riethmacher et al. revealed that neurogenesis of the PNS of SOX10-null mice seemed initially normal, followed by degeneration of motoneurons and sensory neurons during development [47]. It seems that SOX10 ablation did not directly compromise the neuronal differentiation potential of neural crest cells but rather induced cell death of neural cell derivatives. Nevertheless, we found that the neuronal differentiation potential was substantially reduced in the *SOX10* mutant human neural crest cells, and no obvious cell apoptosis could be detected during in vitro neural commitment in either group. These results indicate that SOX10 also plays a crucial role in neuronal differentiation of PNS but is less important in the survival of these neural derivatives. Furthermore, our results also support that SOX10 was required for Schwann cell development and extremely lower number of GFAP^+^ glial cells could be obtained in the SOX10-KO group when compared with WT group. More interestingly, our results reveal that, for the first time, SOX10 deficiency may cause a developmental bias toward nonneural ectodermal cells during neural crest cell induction in vitro according to the results of immunofluorescence staining and RNA-Seq. Whether this phenomenon also occurs in vivo needs further elucidation.

In conclusion, our results suggest that SOX10 plays similar but not the same essential roles in development, migration and differentiation in the human neural crest as in animal models. Consequently, SOX10-knockout hiPSC lines may provide valuable tools for better elucidating *SOX10*gene functions in human neural crest development and*SOX10*-related human neurocristopathies.

## Supplementary information

Fig. S1

Fig. S2

Fig. S3

Fig. S4

Fig. S5

Fig. S6

Fig. S7

Fig. S8

Fig. S9

Fig. S10

Fig. S11

Supplementary Figure Legends

Supplementary Table 1

Supplementary Table 2

Supplementary Table 3

Supplementary Table 4

Supplementary Table 5

## Data Availability

The authors declare that all data supporting the results in this study are available within the paper and its Supplementary Information. The RNA-Seq data have been deposited in the GEO database, under accession number GSE160312.
