# Supplementary figures and images for: SOX10 ablation severely impairs the generation of postmigratory neural crest from human pluripotent stem cells
Source: Cell Death Dis. 2021 Aug 27;12(9):814. doi: 10.1038/s41419-021-04099-4 (PMC8397771; doi:10.1038/s41419-021-04099-4)

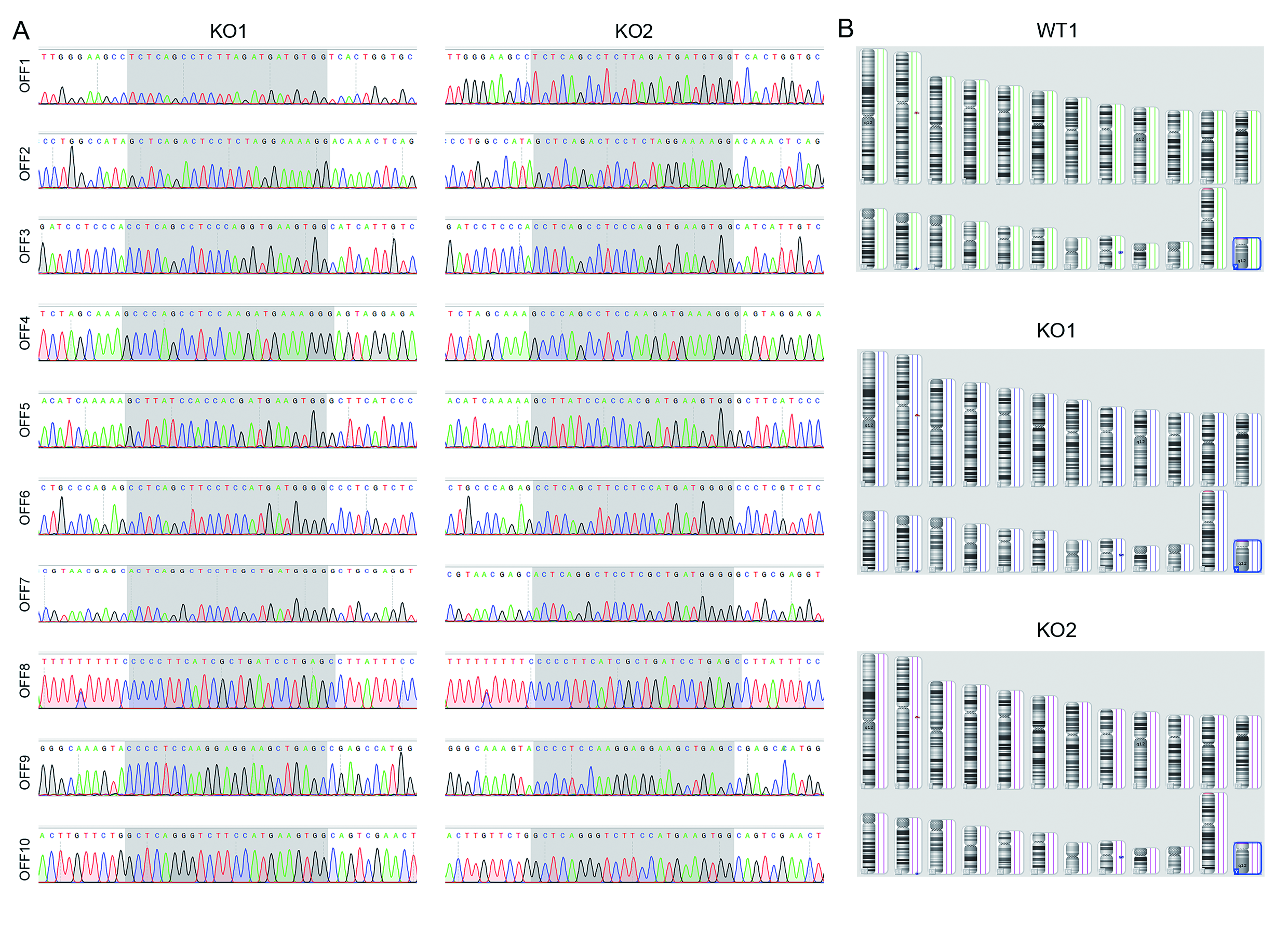

Supplement: Supplementary file 1 — Fig. S1 [file 41419_2021_4099_MOESM1_ESM.tif]

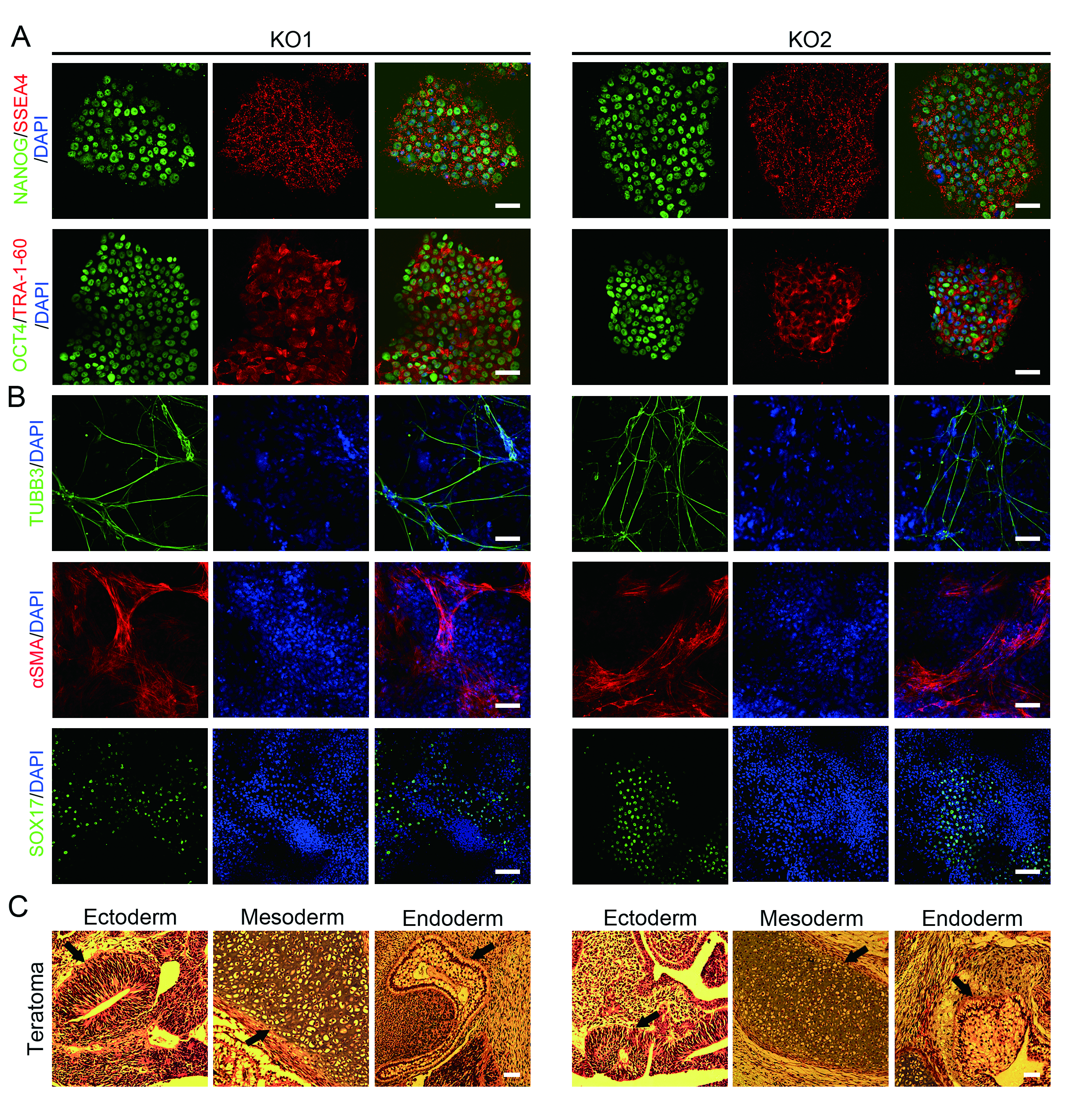

Supplement: Supplementary file 2 — Fig. S2 [file 41419_2021_4099_MOESM2_ESM.tif]

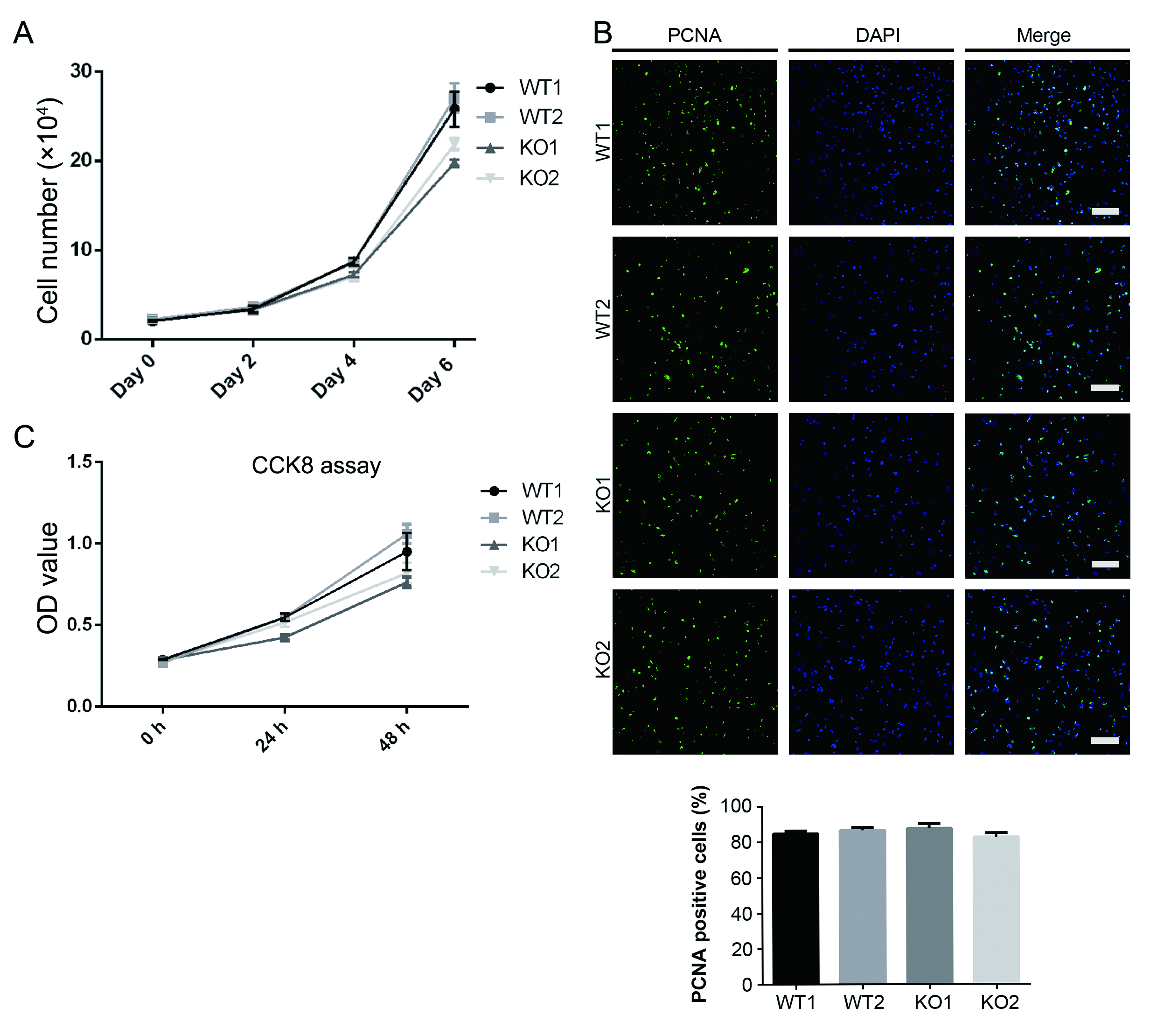

Supplement: Supplementary file 3 — Fig. S3 [file 41419_2021_4099_MOESM3_ESM.tif]

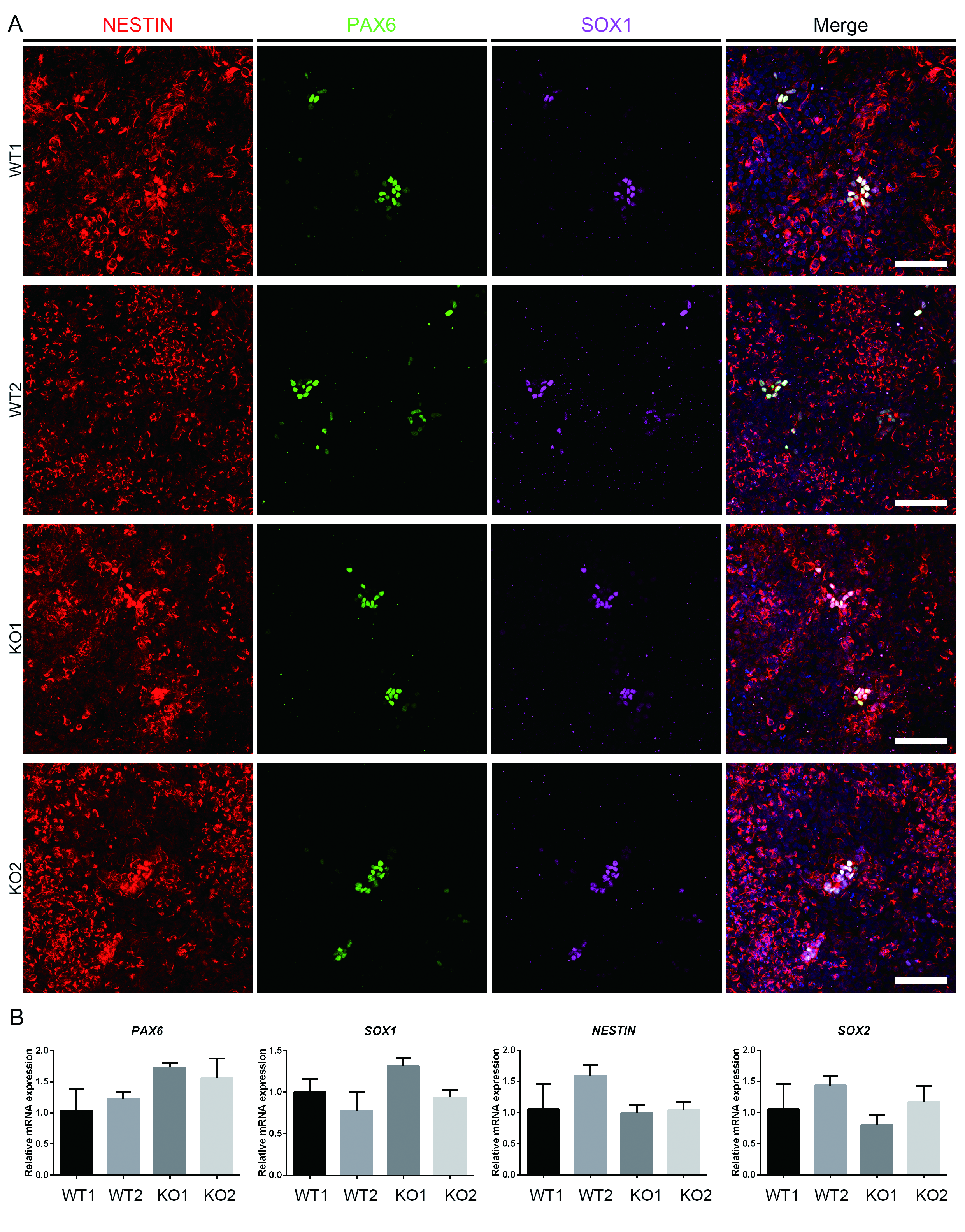

Supplement: Supplementary file 4 — Fig. S4 [file 41419_2021_4099_MOESM4_ESM.tif]

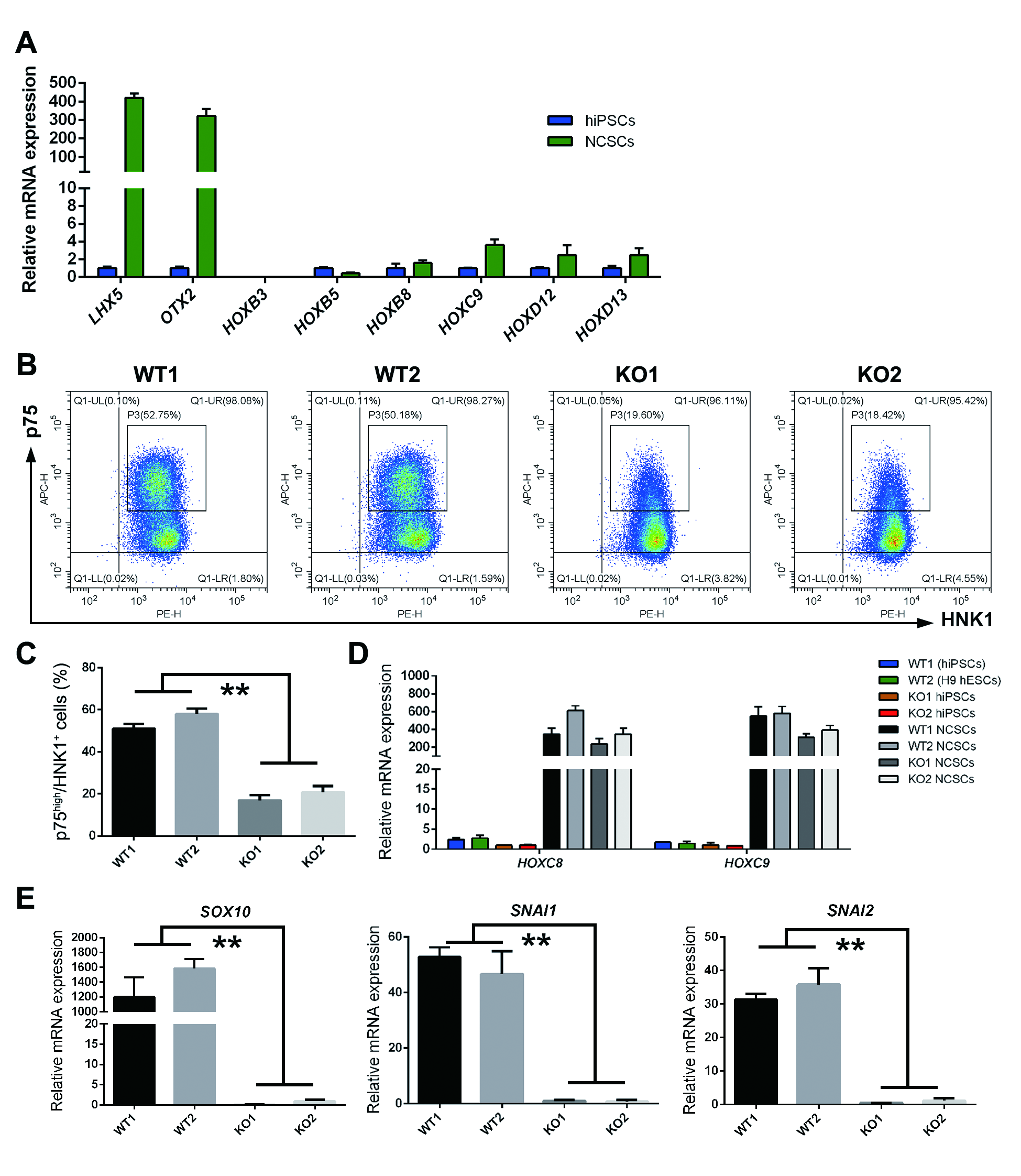

Supplement: Supplementary file 5 — Fig. S5 [file 41419_2021_4099_MOESM5_ESM.tif]

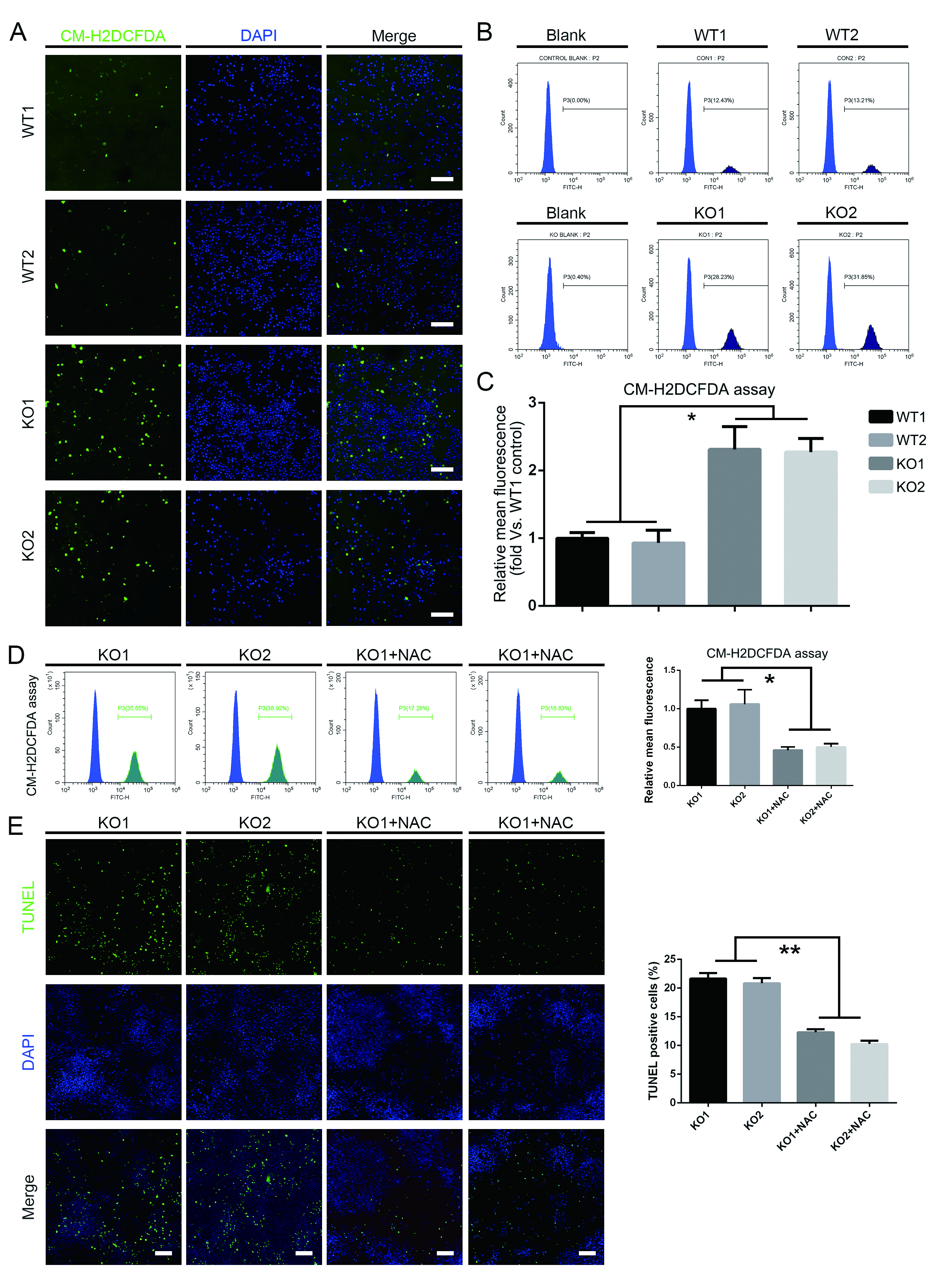

Supplement: Supplementary file 6 — Fig. S6 [file 41419_2021_4099_MOESM6_ESM.tif]

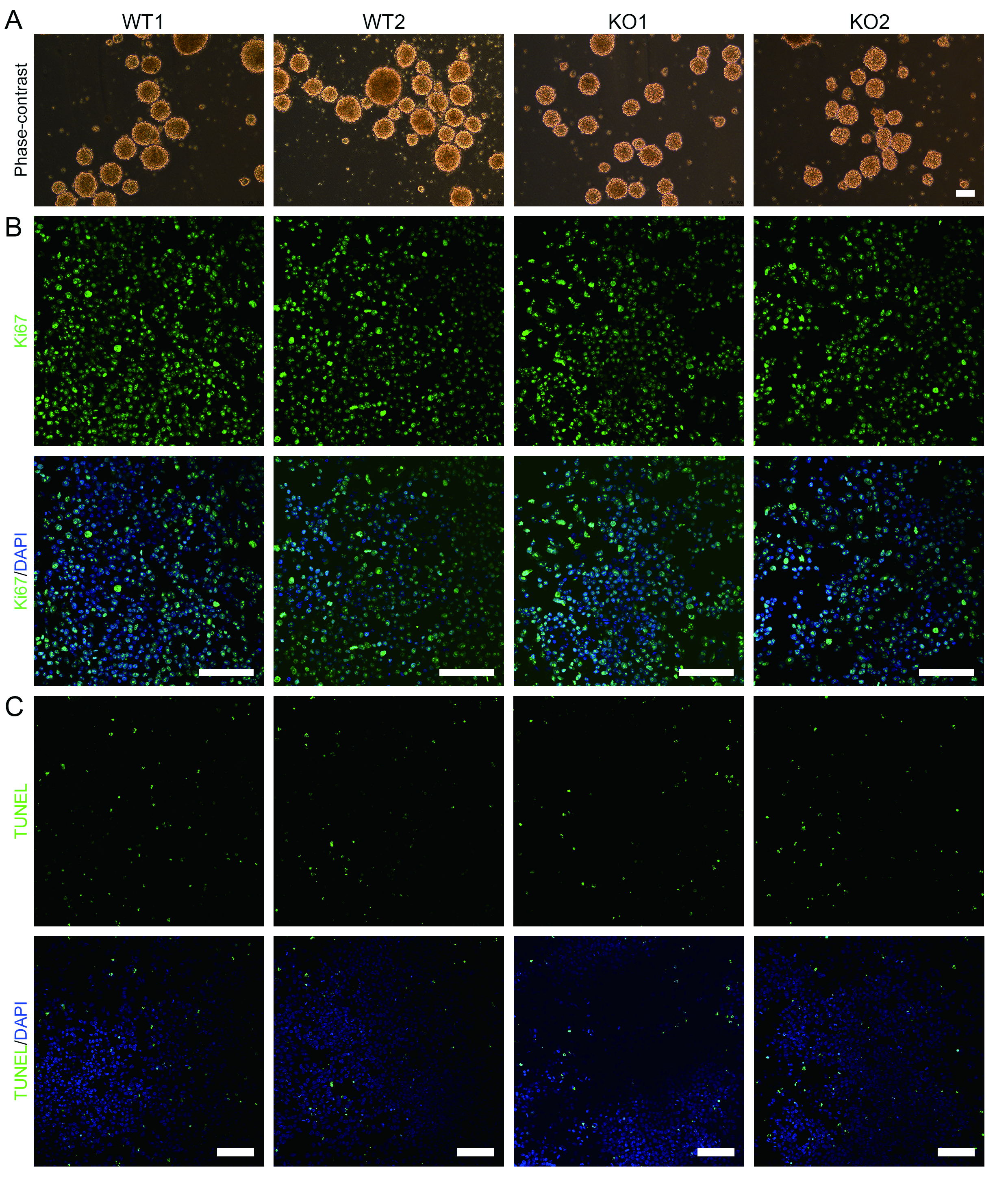

Supplement: Supplementary file 7 — Fig. S7 [file 41419_2021_4099_MOESM7_ESM.tif]

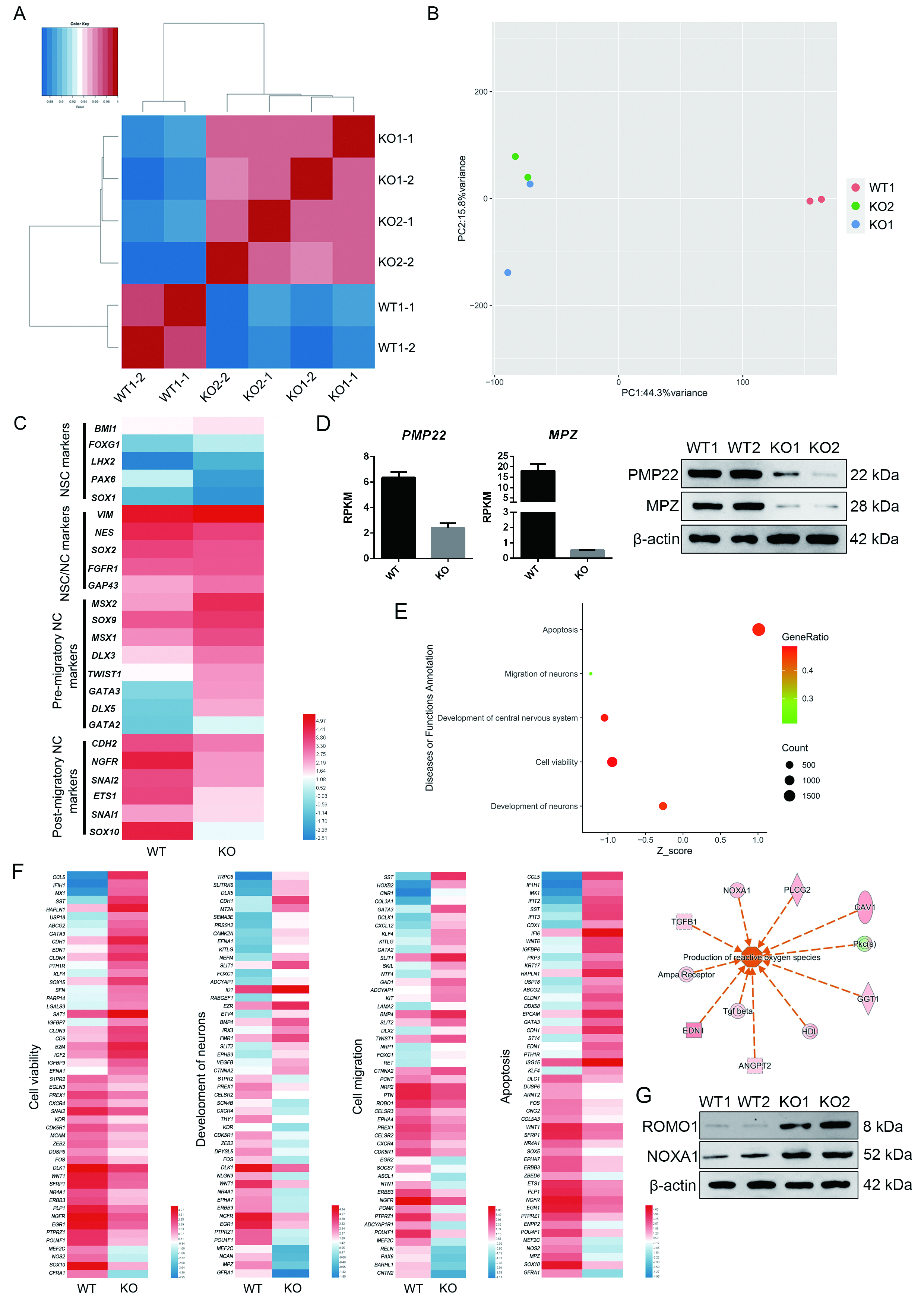

Supplement: Supplementary file 8 — Fig. S8 [file 41419_2021_4099_MOESM8_ESM.tif]

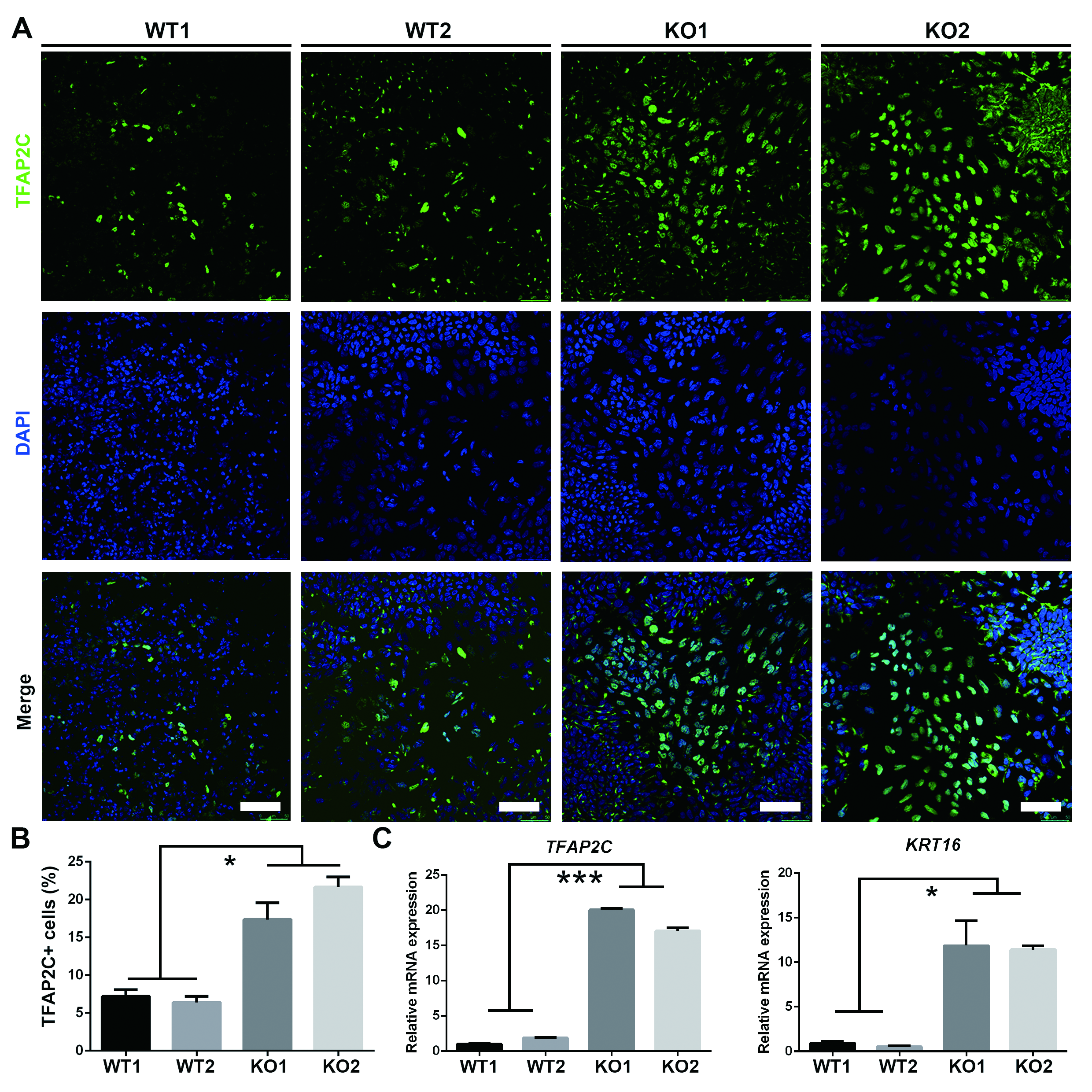

Supplement: Supplementary file 9 — Fig. S9 [file 41419_2021_4099_MOESM9_ESM.tif]

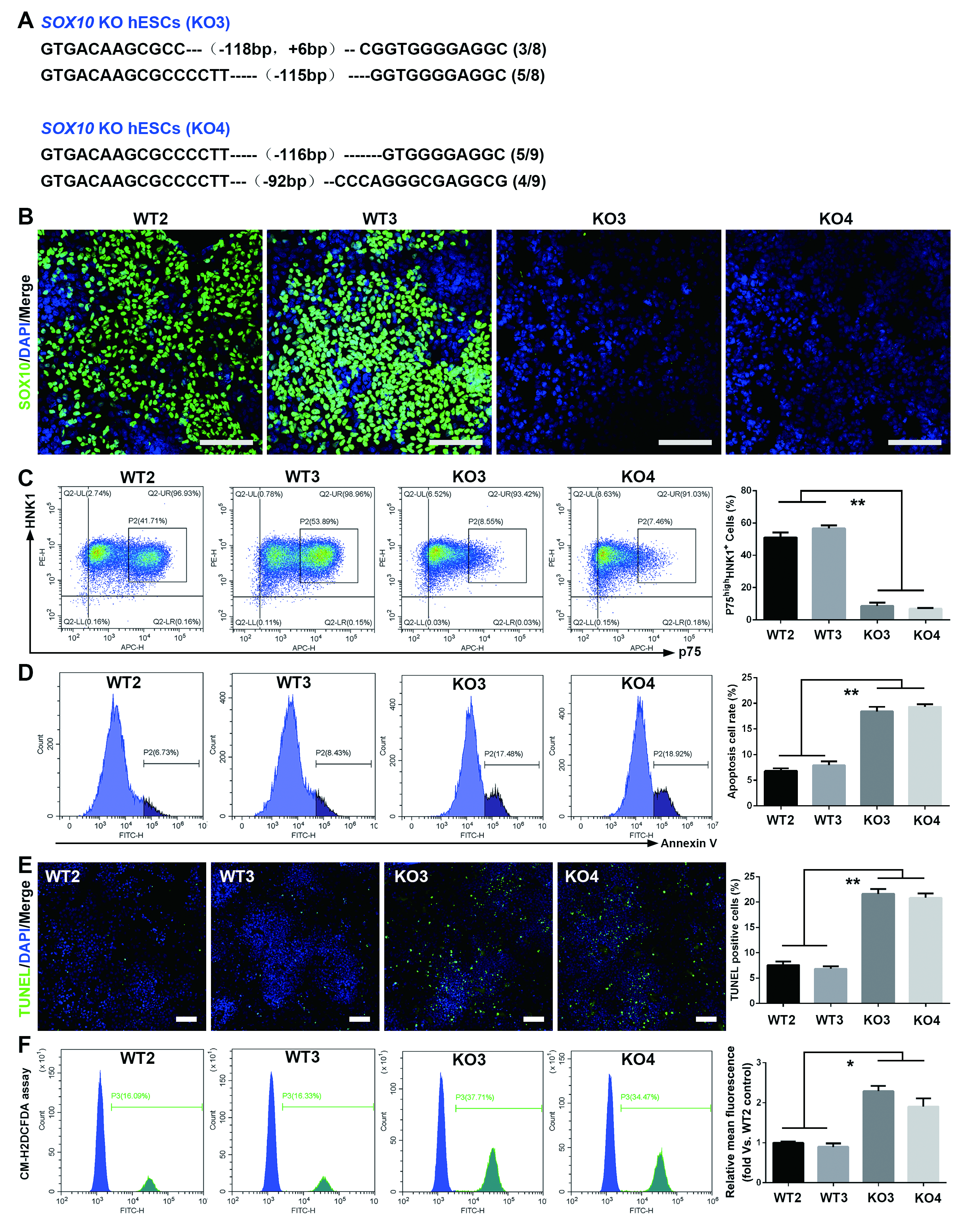

Supplement: Supplementary file 10 — Fig. S10 [file 41419_2021_4099_MOESM10_ESM.tif]

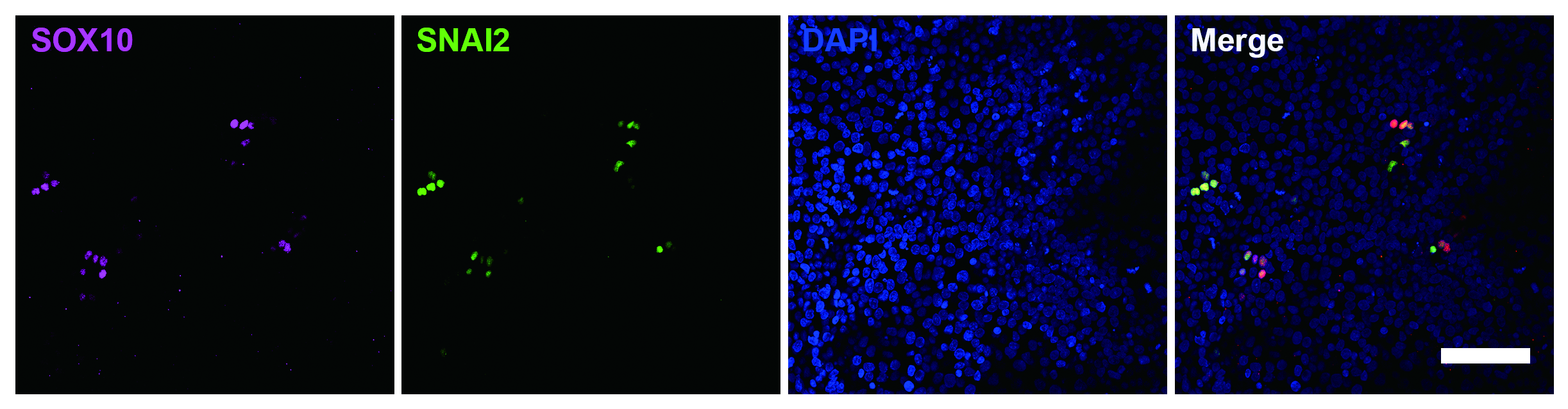

Supplement: Supplementary file 11 — Fig. S11 [file 41419_2021_4099_MOESM11_ESM.tif]
