# Supplementary material for: SOX10 ablation severely impairs the generation of postmigratory neural crest from human pluripotent stem cells
Source: Cell Death Dis. 2021 Aug 27;12(9):814. doi: 10.1038/s41419-021-04099-4 (PMC8397771; doi:10.1038/s41419-021-04099-4)
Supplement: Supplementary file 12 — Supplementary Figure Legends [file 41419_2021_4099_MOESM12_ESM.docx]

**Supplementary Figure Legends**

**Fig. S1 The off-target effects of CRISPR/Cas9-mediated gene editing and the genomic integrity of SOX10-KO hiPSCs were detected.**

A. The top 10 potential off-target sites in SOX10-KO hPSCs were subjected to PCR amplified and Sanger sequencing

B. Chromosomal microarray analysis (CMA) was performed to identify structural chromosomal aberrations in SOX10-KO hiPSCs.

**Fig. S2 Characterization of the KO1 and KO2 hiPSCs.**

A. The expression of pluripotency markers OCT4, NANOG, SSEA4 and TRA-1-60 was detected by immunofluorescence assay. Scale bar, 50 μm.

B. Detection of three germ layer markers in spontaneous differentiated KO1 and KO2 hPSCs in vitro. Scale bar, 50 μm.

C. Detection of three germ layer structures in teratoma formed by KO1 and KO2 hPSCs in vivo. Scale bar, 50 μm.

**Fig. S3 Proliferation assay of differentiated cells from WT and SOX10-KO hiPSCs**

A. Cells were counted and compared at different time points (day 2, day 4 and day 6) during the neural crest differentiation process.

B. The proliferation ability of day 7 cells in WT and KO groups was detected by anti-PCNA immunostaining. Scale bar, 100 μm. PCNA^+^ cells in different groups were counted and compared.

C. The proliferation ability of sphere cells in WT and KO groups was detected by CCK8 assay.

*n* = 5 biological replicates. Columns represent the mean ± SD.

**Fig. S4 Detection of neural epithelial cell markers in day 7 differentiated cells**

A. Expression of neural epithelial cell markers (NESTIN, PAX6, and SOX1) in day 7 differentiated cells in WT and KO groups were detected by immunostaining. Scale bar, 100 μm.

B. The mRNA level of neural epithelial cell markers (*NESTIN*, *PAX6*, *SOX1*, and *SOX2*) in day 7 differentiated cells was detected by qRT-PCR.

*n* = 3 biological replicates. Columns represent the mean ± SD.

**Fig. S5 Trunk neural crest differentiation of WT and SOX10-KO hiPSCs**

A. qRT-PCR analysis was performed to determine which regional type of neural crest cells were generated in our study.

B. The percentage of p75^high^HNK1^+^ postmigratory NCSCs that generated during trunk neural crest differentiation was analyzed by FACS.

C. The percentage of p75^high^HNK1^+^ postmigratory NCSCs was counted and compared.

D. The mRNA levels of trunk markers *HOXC8* and *HOXC9* were detected by qRT-PCR.

E. The mRNA transcripts of postmigratory markers including *SOX10*, *SNAI1* and *SNAI2* were detected by qRT-PCR.

*n* = 3 biological replicates. Columns represent the mean ± SD. ***p* <0.01.

**Fig. S6 Detection of ROS generation in day 7 differentiated cells**

A. The ROS generation was detected by CM-H2DCFDA staining and observed under fluorescence microscope. Scale bar, 100 μm.

B. The ROS generation was detected by CM-H2DCFDA staining and analyzed by FACS.

C. The results of CM-H2DCFDA staining was quantified and compared.

D. The ROS accumulation before and after NAC addition was detected by CM-H2DCFDA staining and analyzed by FACS

E. The cell apoptosis before and after NAC addition was detected by TUNEL assay.

*n* = 3 biological replicates. Columns represent the mean ± SD. **p* <0.05, ***p* <0.01.

**Fig. S7 Characterization of enriched p75^high^/HNK1^+^ NCSCs when cultured in vitro.**

A. Sphere formation of p75^high^/HNK1^+^ NCSCs derived from WT and KO groups. Scale bar, 100 μm.

B. The proliferation ability of p75^high^/HNK1^+^ NCSCs in WT and KO groups was detected by anti-Ki67 immunostaining. Scale bar, 100 μm.

C. The cell apoptosis of p75^high^/HNK1^+^ NCSCs in WT and KO groups was analyzed by TUNEL assay. Scale bar, 100 μm.

**Fig. S8 Global expression profiles of the day 7 differentiated cells**

A. Pearson’s correlation coefficients of pairwise comparisons were calculated for all expressed genes in day 7 differentiated cells between WT and KO groups.

B. Principal component analysis (PCA) was carried out to evaluate the similarities of the gene expression profiles in day 7 differentiated cells between WT and KO groups.

C. The heatmap of makers labeling neural epithelial cells, neural crest/neural epithelial cells, premigratory neural crest, and postmigratory neural crest of day 7 differentiated cells in RNA-Seq results were analyzed, respectively.

D. The expression of SOX10 target genes (PMP22 and MPZ) was validated by western blotting.

E. The expression levels of genes involved in cell survival, migration, neural development, apoptosis and ROS generation of day 7 differentiated cells in RNA-Seq results (the selected set of genes with RPKM value ≥ 5) were analyzed by Ingenuity Pathways Analysis (IPA) software.

F. The heatmaps of genes involved in cell survival, migration, neural development, apoptosis and ROS generation of day 7 differentiated cells were presented, respectively.

G. The expression of ROS pathway members (NOXA1 and ROMO1) was validated by western blotting.

**Fig. S9 Detection of NNE cells within day 7 differentiated cell population.**

A. Expression of NNE marker TFAP2C in day 7 differentiated cells was detected by immunostaining. Scale bar, 50 μm.

B. TFAP2C positive cells were counted and compared. *n* = 5 per group. Columns represent the mean ± SD. **p* <0.05.

C. The mRNA level of NNE marker *TFAP2C* and *KRT16* in day 7 differentiated cells was detected by qRT-PCR.

*n* = 3 biological replicates. Columns represent the mean ± SD. **p* <0.05, ****p* <0.001.

**Fig. S10 Verification and neural crest differentiation of SOX10-knockout hESCs.**

A. Sanger sequencing was performed on PCR products amplifed from SOX10-KO hESCs;

B. The protein expression level of SOX10 in WT and SOX10-KO hESCs was detected by immunofluorescence assay (Scale bar: 100μm);

C. The proportion of p75high/HNK1+ migrating NCSCs in day 7 differentiated cells of WT and KO hESCs was analyzed by FACS (**p < 0.01);

D. The cell apoptosis in day 7 cell cultures in different groups was analyzed by Annexin V staining (**p < 0.01);

E. The cell apoptosis in day 7 cell cultures in different groups was analyzed by TUNEL assay (**p < 0.01; Scale bar: 100μm);

F. The ROS generation was detected by CM-H2DCFDA staining and analyzed by FACS (*p < 0.05).

**Fig. S11 Immunostaining of day 5 differentiated cells with anti-SOX10 and anti-SNAI2 antibodies** (Scale bar: 100μm)
