# Supplementary material for: SOX10 ablation severely impairs the generation of postmigratory neural crest from human pluripotent stem cells
Source: Cell Death Dis. 2021 Aug 27;12(9):814. doi: 10.1038/s41419-021-04099-4 (PMC8397771; doi:10.1038/s41419-021-04099-4)
Supplement: Supplementary file 14 — Supplementary Table 2 [file 41419_2021_4099_MOESM14_ESM.docx]

**Supplementary Table 2. Primers used for qRT-PCR**

| **Gene** | **Direction** | **Sequences** |
| --- | --- | --- |
| *ACTB* | Forward | 5’ CAT GTA CGT TGC TAT CCA GGC 3’ |
|  | Reverse | 5’ CTC CTT AAT GTC ACG CAC GAT 3’ |
| *CDH1* | Forward | 5’ CGA GAG CTA CAC GTT CAC GG 3’ |
|  | Reverse | 5’ GGG TGT CGA GGG AAA AAT AGG 3’ |
| *CDH2* | Forward | 5’ TCA GGC GTC TGT AGA GGC TT 3’ |
|  | Reverse | 5’ ATG CAC ATC CTT CGA TAA GAC TG 3’ |
| *DLX3* | Forward | 5’ TAC CCT GCC CGA GTC TTC TG 3’ |
|  | Reverse | 5’ TGG TGG TAG GTG TAG GGG TTC 3’ |
| *DLX5* | Forward | 5’ TTC CAA GCT CCG TTC CAG AC 3’ |
|  | Reverse | 5’ GAA TCG GTA GCT GAA GAC TCG 3’ |
| *ETS1* | Forward | 5’ TAC ACA GGC AGT GGA CCA ATC 3’ |
|  | Reverse | 5’ CCC CGC TGT CTT GTG GAT G 3’ |
| *GAPDH* | Forward | 5’ CCC CTT CAT TGA CCT CAA CTA CA 3’ |
|  | Reverse | 5’ TTG CTG ATG ATC TTG AGG CTG T 3’ |
| *GFAP* | Forward | 5’ CTG CGG CTC GAT CAA CTC A 3’ |
|  | Reverse | 5’ TCC AGC GAC TCA ATC TTC CTC 3’ |
| *KRT16* | Forward | 5’ GAC CGG CGG AGA TGT GAA C 3’ |
|  | Reverse | 5’ CTG CTC GTA CTG GTC ACG C 3’ |
| *MSX1* | Forward | 5’ ACC TCT TTG CTC CCT GAG TTC AC 3’ |
|  | Reverse | 5’ GAC TCT TCC AGC CAC TTT TTG G 3’ |
| *MSX2* | Forward | 5’ ATG GCT TCT CCG TCC AAA GG 3’ |
|  | Reverse | 5’ CGG CTT CTT GTC GGA CAT GA 3’ |
| *NESTIN* | Forward | 5’ AAC AGC GAC GGA GGT CTC TA 3’ |
|  | Reverse | 5’ TTC TCT TGT CCC GCA GAC TT 3’ |
| *P75* | Forward | 5’ CTG CCT GGA CAG CGT GAC GTT 3’ |
|  | Reverse | 5’ GCA GCG CCC AGT CGT CTC AT 3’ |
| *PAX6* | Forward | 5’ AAC GAT AAC ATA CCA AGC GTG T 3’ |
|  | Reverse | 5’ GGT CTG CCC GTT CAA CAT C 3’ |
| *PRPH* | Forward | 5’ GCC TGG AAC TAG AGC GCA AG 3’ |
|  | Reverse | 5’ CCT CGC ACG TTA GAC TCT GG 3’ |
| *S100B* | Forward | 5’ TGG CCC TCA TCG ACG TTT TC 3’ |
|  | Reverse | 5’ ATG TTC AAA GAA CTC GTG GCA 3’ |
| *SOX1* | Forward | 5’ CAG TAC AGC CCC ATC TCC AAC 3’ |
|  | Reverse | 5’ GCGGGCAAGTACATGCTGA 3’ |
| *SOX2* | Forward | 5’ GGG AAA TGG GAG GGG TGC AAA AGA GG 3’ |
|  | Reverse | 5’ TTG CGT GAG TGT GGA TGG GAT TGG TG 3’ |
| *SOX9* | Forward | 5’ AGC GAA CGC ACA TCA AGA C 3’ |
|  | Reverse | 5’ CTG TAG GCG ATC TGT TGG GG 3’ |
| *SOX10* | Forward | 5’ CCC GCA CTA CAC CGA CCA 3’ |
|  | Reverse | 5’ AGG AGA AAG CCG AGT AGA 3’ |
| *SNAI1* | Forward | 5’ TCG GAA GCC TAA CTA CAG CGA 3’ |
|  | Reverse | 5’ AGA TGA GCA TTG GCA GCG AG 3’ |
| *SNAI2* | Forward | 5’ CGA ACT GGA CAC ACA TAC AGT G 3’ |
|  | Reverse | 5’ CTG AGG ATC TCT GGT TGT GGT 3’ |
| *TFAP2A* | Forward | 5’ GTA AAG CTG CCA ACG TTA CCC TC 3’ |
|  | Reverse | 5’ TTG GCA GGA AAT TCG GTT TCA CAC 3’ |
| *TUBB3* | Forward | 5’ GGC CAA GGG TCA CTA CAC G 3’ |
|  | Reverse | 5’ GCA GTC GCA GTT TTC ACA CTC 3’ |
| *TWIST1* | Forward | 5’ GTC CGC AGT CTT ACG AGG AG 3’ |
|  | Reverse | 5’ GCT TGA GGG TCT GAA TCT TGC T 3’ |
| *HOXB4* | Forward | 5’ AAA GAG CCC GTC GTC TAC C 3’ |
|  | Reverse | 5’ GTG TAG GCG GTC CGA GAG 3’ |
| *HOXC4* | Forward | 5’ GTC GCT AGC TAG TAG GAG GG 3’ |
|  | Reverse | 5’ TCC GGA CTG TGT TCA GGG AT 3’ |
| *HOXC5* | Forward | 5’ ACA GAT TTA CCC GTG GAT GAC 3’ |
|  | Reverse | 5’ AGT GAG GTA GCG GTT AAA GTG 3’ |
| *HOXC6* | Forward | 5’ GAA TGA GGG AAG ACG AGA AAG A 3’ |
|  | Reverse | 5’ CAT AGG CGG TGG AAT TGA GG 3’ |
| *HOXB8* | Forward | 5’ AGC CTC CTT GTG CAA TTG 3’ |
|  | Reverse | 5’ GTA ACA ATT GCC CAC AGC 3’ |
| *HOXC8* | Forward | 5’ CCT CCG CCA ACA CTA ACA GT 3’ |
|  | Reverse | 5’ CCA AGG TCT GAT ACC GGC TG 3’ |
| *HOXC9* | Forward | 5’ GGG AGG GTT CAG TGT TGA GA 3’ |
|  | Reverse | 5’ GGG ATG ACC TGG ACC AAA TA 3’ |
| *HOXD12* | Forward | 5’ CTC TCA AAG CGG CCA AGT ATG 3’ |
|  | Reverse | 5’ CTG CTT CGT GTA GGG TTT CC 3’ |
| *HOXD13* | Forward | 5’ CTT CGG CAA CGG CTA CTA CAG 3’ |
|  | Reverse | 5’ TGA CAC GTC CAT GTA CTT CTC C 3’ |
