# Supplementary material for: SOX10 ablation severely impairs the generation of postmigratory neural crest from human pluripotent stem cells
Source: Cell Death Dis. 2021 Aug 27;12(9):814. doi: 10.1038/s41419-021-04099-4 (PMC8397771; doi:10.1038/s41419-021-04099-4)
Supplement: Supplementary file 15 — Supplementary Table 3 [file 41419_2021_4099_MOESM15_ESM.docx]

**Supplementary Table 3. Antibodies used in Western blotting**

| **Antigen** | **Host** | **Company** | **Cat. No.** |
| --- | --- | --- | --- |
| β-actin | mouse | Bioss | bsm-33036M |
| MPZ | rabbit | abcam | ab180933 |
| NOXA1 | rabbit | Abnova | H00010811-D01 |
| PMP22 | mouse | Santa cruz | sc-65739 |
| ROMO1 | rabbit | Signalway Antibody | 47598 |
| SOX10 | rabbit | abcam | ab155279 |
| GAPDH | rabbit | Cell Signaling Technology | 2118s |
| anti-mouse IgG HRP-linked Ab | goat | Cell Signaling Technology | 7056 |
| anti-rabbit IgG HRP-linked Ab | goat | Cell Signaling Technology | 7074 |
