# Supplementary material for: SOX10 ablation severely impairs the generation of postmigratory neural crest from human pluripotent stem cells
Source: Cell Death Dis. 2021 Aug 27;12(9):814. doi: 10.1038/s41419-021-04099-4 (PMC8397771; doi:10.1038/s41419-021-04099-4)
Supplement: Supplementary file 16 — Supplementary Table 4 [file 41419_2021_4099_MOESM16_ESM.docx]

**Supplementary Table 4. Antibodies used in FACS**

| Antigen | Label | Company | Cat. No. |
| --- | --- | --- | --- |
| Anti-human CD271 (p75) | AF647 | BD Pharmingen | 560326 |
| Anti-human CD271 (p75) | PE | BD Pharmingen | 557196 |
| Anti-human CD49d | APC | BD Pharmingen | 559881 |
| Anti-human CD57 (HNK1) | PE | BD Pharmingen | 560844 |
