# Supplementary material for: SOX10 ablation severely impairs the generation of postmigratory neural crest from human pluripotent stem cells
Source: Cell Death Dis. 2021 Aug 27;12(9):814. doi: 10.1038/s41419-021-04099-4 (PMC8397771; doi:10.1038/s41419-021-04099-4)
Supplement: Supplementary file 17 — Supplementary Table 5 [file 41419_2021_4099_MOESM17_ESM.docx]

**Supplementary Table 5. Antibodies used in Immunofluorescence Staining**

| **Antigen** | **Host** | **Dilution** | **Company** | **Cat. No.** |
| --- | --- | --- | --- | --- |
| α-SMA | mouse | 1:500 | Abcam | ab7817 |
| GFAP | rabbit | 1:500 | Millipore | ab5804 |
| HNK1 | mouse | 1:200 | Sigma-Aldrich | c6680 |
| Ki67 | rabbit | 1:200 | Abcam | ab15580 |
| MSX1 | goat | 1:200 | R&D System | AF5045 |
| Nanog | rabbit | 1:200 | Cell signaling technology | 3580 |
| Nestin | mouse | 1:200 | EMD Millipore | MAB5326 |
| Oct3/4 | rabbit | 1:200 | Santa cruz | Sc-9081 |
| PAX6 | rabbit | 1:200 | Abcam | ab195045 |
| PCNA | mouse | 1:200 | Abcam | ab29 |
| Peripherin | rabbit | 1:200 | Abcam | Ab123576 |
| p75 | rabbit | 1:200 | Promega | G3231 |
| p75 | mouse | 1:200 | Thermo Fisher Scientific | MA5-13314 |
| SLUG | rabbit | 1:100 | Abcam | ab27568 |
| SSEA-4 | mouse | 1:200 | EMD Millipore | MAB4304 |
| SOX1 | goat | 1:200 | R&D System | AF3369 |
| SOX9 | rabbit | 1:200 | EMD Millipore | AB5535 |
| SOX10 | rabbit | 1:200 | Abcam | ab155279 |
| SOX10 | goat | 1:100 | Thermo Fisher Scientific | PA5-47001 |
| SOX17 | goat | 1:100 | R&D System | AF1924 |
| TFAP2C | rabbit | 1:200 | Sigma-Aldrich | HPA055179 |
| TRA-1-60 | mouse | 1:200 | EMD Millipore | MAB4360 |
| Tuj1 | mouse | 1:500 | R&D System | MAB1195 |
| Goat anti-mouse IgG Alexa 488 | goat | 1:1000 | Invitrogen | A11001 |
| Goat anti-rabbit IgG Alexa 488 | goat | 1:1000 | Invitrogen | A11008 |
| Goat anti-rabbit IgG Alexa 555 | goat | 1:1000 | Invitrogen | A21428 |
| Goat anti-mouse IgG Alexa 555 | goat | 1:1000 | Invitrogen | A21422 |
| Donkey anti-goat IgG Alexa 594 | donkey | 1:1000 | Invitrogen | A11058 |
